# The role and impact of alternative polyadenylation and miRNA regulation on the expression of the multidrug resistance-associated protein 1 (MRP-1/ABCC1) in epithelial ovarian cancer

**DOI:** 10.1038/s41598-023-44548-y

**Published:** 2023-10-14

**Authors:** Audrey Marjamaa, Bettine Gibbs, Chloe Kotrba, Chioniso Patience Masamha

**Affiliations:** 1 Department of Chemistry and Biochemistry, Butler University, Indianapolis, IN 46208 USA; 2 Department of Pharmaceutical Sciences, Butler University, Indianapolis, IN 46208 USA; 3 Department of Microbiology, Harvard Medical School, Boston, MA 02115 USA

**Keywords:** Alternative splicing, RNAi, Gene expression, Ovarian cancer, Cell death, Molecular medicine

## Abstract

The ATP-binding cassette transporter (*ABCC1)* is associated with poor survival and chemotherapy drug resistance in high grade serous ovarian cancer (HGSOC). The mechanisms driving *ABCC1* expression are poorly understood. Alternative polyadenylation (APA) can give rise to *ABCC1* mRNAs which differ only in the length of their 3′untranslated regions (3′UTRs) in a process known as 3′UTR-APA. Like other ABC transporters, shortening of the 3′UTR of *ABCC1* through 3′UTR-APA would eliminate microRNA binding sites found within the longer 3′UTRs, hence eliminating miRNA regulation and altering gene expression. We found that the HGSOC cell lines Caov-3 and Ovcar-3 express higher levels of *ABCC1* protein than normal cells. APA of *ABCC1* occurs in all three cell lines resulting in mRNAs with both short and long 3′UTRs. In Ovcar-3, mRNAs with shorter 3′UTRs dominate resulting in a six-fold increase in protein expression. We were able to show that miR-185-5p and miR-326 both target the *ABCC1* 3′UTR. Hence, 3′UTR-APA should be considered as an important regulator of *ABCC1* expression in HGSOC. Both HGSOC cell lines are cisplatin resistant, and we used erastin to induce ferroptosis, an alternative form of cell death. We showed that we could induce ferroptosis and sensitize the cisplatin resistant cells to cisplatin by using erastin. Knocking down *ABCC1* resulted in decreased cell viability, but did not contribute to erastin induced ferroptosis.

## Introduction

Although ovarian cancer makes up only three percent of all cancer cases in women, it remains the fifth leading cause of cancer death in women in the United States and the leading cause of death for all gynecologic malignancies^1^. High-grade serous ovarian cancer (HGSOC), accounts for two-thirds of these deaths and is considered the most prevalent and aggressive epithelial ovarian cancer subtype^2,3^. Despite aggressive treatment, the five-year survival rate in these patients is only 30%, with most deaths resulting from relapsed, drug-resistant disease^4^. The development of chemotherapy resistance is multifactorial and includes alterations in DNA damage repair pathways, decreased apoptosis, and increased activity of ATP‐binding cassette (ABC) drug efflux transporters^5^. The overexpression of three ABC transporters in particular, breast cancer resistance protein (*BCRP/MXR/ABCG2)*, P-glycoprotein (P-gp/*MDR-1-/ABCB1),* and multi-drug resistance associated protein 1 (*MRP-1/ABCC1),* are associated with increased multi-drug resistance and shorter progression free survival in ovarian cancer^6,7^. In some cases, there is conflicting information on the actual mRNA levels detected in tumors^8,9^. For some transporters, the levels of mRNA do not correspond to the increased levels of protein detected in tumor samples^9,10^. Determining the mechanisms that regulate the expression of these drug transporters in cancer is critical.

Alternative polyadenylation (APA), a form of post-transcriptional regulation which generates mRNAs with different 3′untranslated regions (3′UTRs) during 3′end formation, is increasingly gaining importance as a mechanism that fine-tunes gene expression^11–13^. Non-histone coding mRNAs undergo 3′end formation, which involves 3′end cleavage usually ~ 15 nucleotides downstream of a polyadenylation signal (PAS), and the addition of adenines to generate polyadenylated mRNAs^14,15^. However, almost three-quarters of human genes have multiple PASs and 3′end formation can occur at alternative sites through APA^11^. APA may involve splicing thus generating different protein products. More commonly, APA may generate mRNAs which code for the same protein product but differ in the length of their 3′UTRs (3′UTR-APA)^16^. Different lengths of the 3′UTR may affect the function of the mRNA as well as its metabolism due in part to differences in sequence content^17–20^. Shortening of the 3′UTR eliminates miRNA and RNA binding sites found in the longer 3′UTRs. In ovarian cancer, several miRNAs are associated with resistance to either cisplatin (miR-214, Let-7i) or paclitaxel (miR-663 and miR-622). The miRNA (miR-130b) which is associated with Pg-p regulation is known to be involved in both cisplatin and paclitaxel multi-drug resistance^21^. Recent work suggests that treatment with cisplatin induces shortening of the 3′UTR of genes involved in DNA damage response, the cell cycle, and apoptosis, preceding these cellular processes by seven hours^22^. Hence, post-transcriptional regulation through 3′UTR-APA may play a crucial role in the expression of genes involved in cisplatin resistance in HGSOC.

Due to innate or acquired chemoresistance, there has been great interest in trying to target ABC transporters for anti-cancer treatment. Out of all the ABC transporters, P-gp (*ABCB1)* which is often detected in tumors with the multidrug resistant phenotype, is the most well-known and has been the major target for many therapies^9,23^ . However, all the first to third-generation drugs that have been developed to target Pg-p protein have had disappointing results in clinical trials^10^. Despite Pg-p’s dominance in the field, the role of *ABCC1* in the efflux and thus drug resistance to traditional chemotherapeutic agents, as well as the newer targeted therapies including small-molecule tyrosine kinase inhibitors, is increasing its prominence in the field^23^. Several *ABCC1* targeted monoclonal antibodies that interfere with substrate binding and efflux were developed for experimental use^24^. The *ABCC1* inhibitors Reversan and MK571 have been shown to inhibit the growth of prostate cancer cell lines^25^. Use of RNA interference to specifically target the *ABCC1* drug transporter is a potential avenue for targeted therapy. There is also an increased interest in using dual and multi-targeted ABC inhibitors to sensitize tumor cells to chemotherapy^26,27^. This multi-targeted approach may address emerging research that suggests that that targeted inhibition of one ABC transporter may result in induced upregulation of a different ABC transporter to compensate^28^.

Another approach to target chemotherapy resistance has been sensitizing tumor cells to apoptosis or trying to induce alternative forms of cell death including autophagy and ferroptosis^29^. Ferroptosis is defined as a regulated form of iron dependent cell death resulting from oxidative stress within the intracellular microenvironment, which is constitutively controlled by reduced glutathione (GSH)-dependent enzyme glutathione peroxidase 4 (GPX4)^29^. Interestingly, *ABCC1* can bind to GSH although the significance of this binding to ferroptosis is still unknown^23^. Ferroptosis can be molecularly inhibited by lipophilic antioxidants and iron chelators^29^. A study showed that patients with HGSOC had lower levels of the iron efflux pump ferroportin (*FPN*) and higher levels of the iron importer transferrin receptor (*TFR1*) when compared to patients with low grade serous ovarian cancer^30^. High levels of iron and malondialdehyde were also observed in ovarian cancer^31^. These changes in *TFR1* and *FPN* and the accompanying iron accumulation made the cells more susceptible to ferroptosis, which can be exploited therapeutically^30,31^. The small molecule, erastin which induces ferroptosis, has been previously reported to enhance efficacy of chemotherapy in glioblastoma and non-small cell lung cancer cells^32,33^. A study using the non-HGSOC ovarian cancer cell line A2780, showed that treatment with erastin together with docetaxel resulted in increased apoptosis in *ABCB1* over-expressing A2780 cells. Erastin was posited to restrict drug-efflux activity through an unknown mechanism^34^.

The focus of this study was to determine the protein and mRNA expression of the *ABCC1* drug transporter in HGSOC cell lines and to investigate the potential role of 3′UTR-APA in generating different *ABCC1* transcripts and miRNA evasion. The effects of erastin and *ABCC1* depletion on inducing ferroptosis were also determined.

## Results

### Analysis of *ABCC1* expression in HGSOC cells

**Figure 1:**
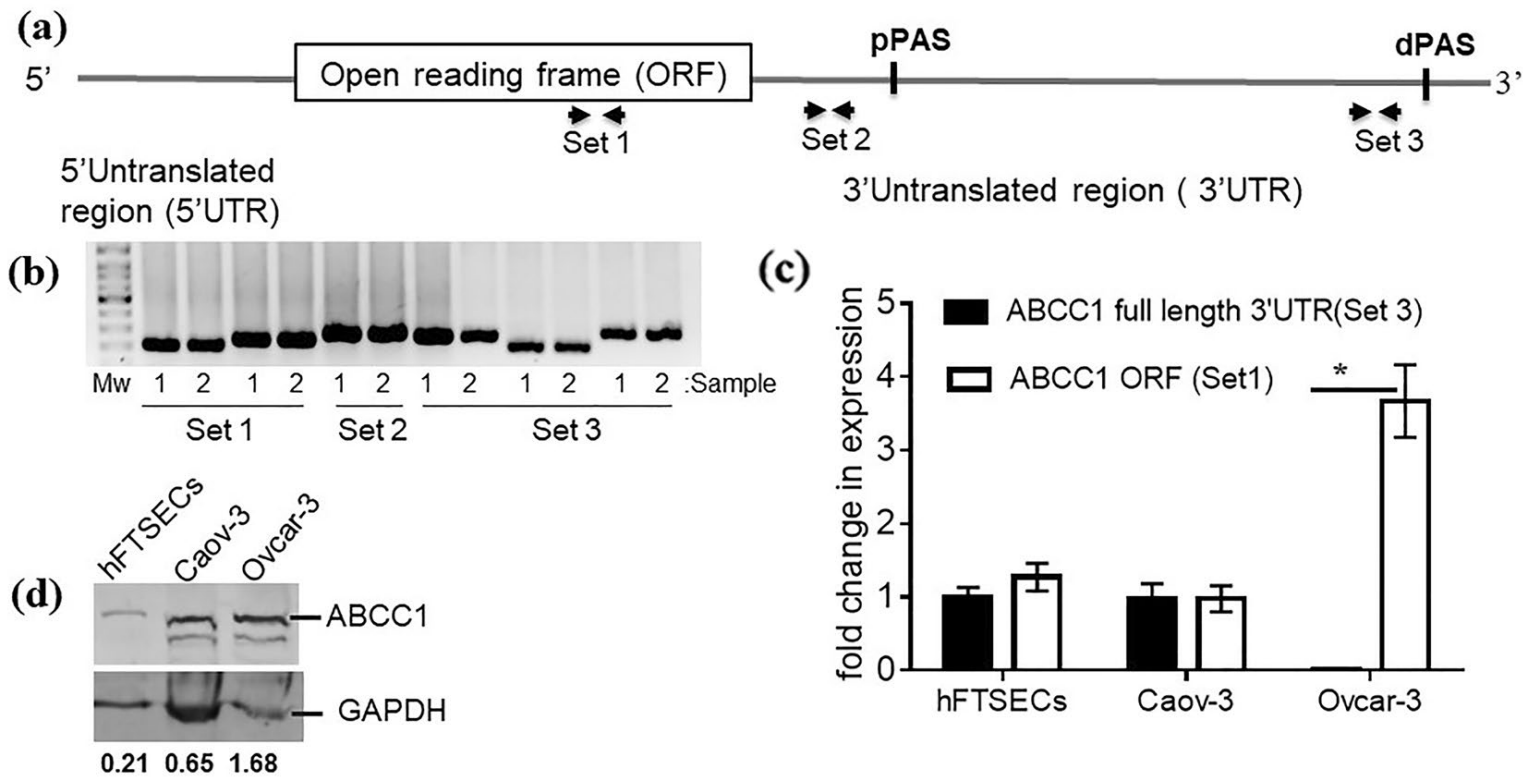
Determination of *ABCC1* expression in ovarian cancer. (**a**) A schematic showing the approximate location of different sets of primers designed to detect *ABCC1*. (**b**) An Ethidium bromide stained gel showing the PCR product of different primer combinations for *ABCC1*. The PCRs were done in biological duplicates (sample 1 and 2) for different primer sets. (**c**) Quantitative real-time PCR (qRT-PCR) results on RNA from human fallopian tube fimbria secretory epithelial cells (hFTSECs) and two epithelial ovarian cancer cell lines Caov-3 and Ovcar-3 using primers located in the open reading frame (ORF) and the long 3′UTR. The expression levels were normalized to GAPDH and are relative to the levels of the expression of the full length 3’UTR in hFTSECs using the 2^–∆∆Ct^ method. Shown is the average (n = 3,  ± s.d. with *p < 0.001). (**d**) Western blot of cell lysates from hFTSECs and the Caov-3 and Ovcar-3 cell lines probed for *ABCC1* protein and GAPDH. Densitometric readings for *ABCC1* each lane was normalized to those of GAPDH using Image J and the values are shown below the Western Blot.

Multiple primers within the ORF and the 3′UTR of *ABCC1* were designed for use in the PCR (Fig. 1a). A combination of different sets of primers was used to identify primers for potential use in subsequent experiments. For screening primers, we used RNA derived from the frequently used, non-HGSOC ovarian cancer cell line Skov-3^1,35^ as our testing model (Fig. 1b). To determine the levels of short and long *ABCC1* mRNAs arising from 3′UTR-APA, two sets of forward and reverse primers were selected for qRT-PCR. One set was located within the open reading frame (set 1) and another within the long 3′UTR (Set 3) (Fig. 1a). After RNA extraction, qRT-PCR was carried out on two cell lines Caov-3 and Ovcar-3 which were genomically validated as highly likely HGSOC (Fig. 1c)^1,35^. The qRT-PCR results were normalized to levels of *ABCC1* mRNA in what is currently hypothesized as the cell of origin for HGSOC, human fallopian tube secretory epithelial cells (hFTSECs)^36–39^. The levels of *ABCC1* mRNA detected by both sets of primers in hFTSECs and Caov-3 cells were similar suggesting that the cell lines express equal levels of transcripts with short and full length 3′UTRs(Fig. 1c). However, in the Ovcar-3 cell line there was a significant decrease in the levels of transcripts with the long 3′UTRs and an increase in transcripts with the shorter 3′UTR. Western blot analysis showed that both HGSOC cell lines expressed higher levels of *ABCC1* when compared to hFTSECs (Fig. 1d). The full length blot (Supplementary Fig. 1a) and the densitometric readings for this image are provided (Supplementary Fig. 1b). However, Ovcar-3 cells expressed higher levels of *ABCC1* protein than Caov-3 cells.

### Characterization of the different *ABCC1* 3′UTR sequences in HGSOC

**Figure 2:**
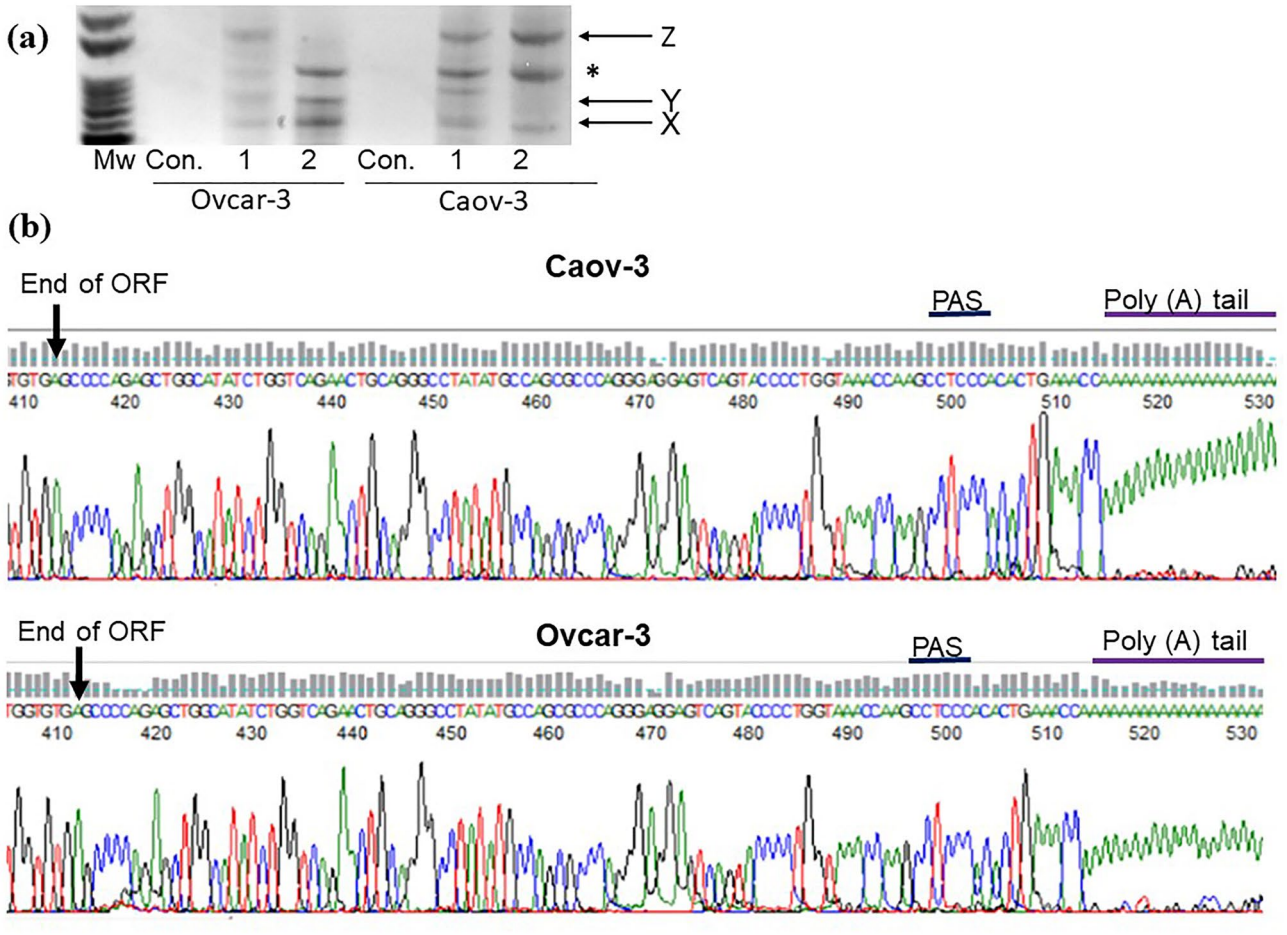
*ABCC1* 3′UTR sequences in ovarian cancer. (**a**) 3′RACE second PCR products from ovarian cancer cells run on an ethidium bromide-stained agarose gel. Shown are results from two biological replicates (1 and 2) and the no cDNA control (Con.) (**b**) Sequence chromatogram showing the TGA codon making up the end of the open reading frame (ORF), the polyadenylation signal (PAS) and the poly(A) tail in the 3′untranslated region (3′UTR) from the shortest 3′RACE product (product X from panel **a**).

In order to identify the optimal primers to detect different 3′UTRs from *ABCC1,* we performed 3′RACE using nested forward primers located within the ORF (Fig. 1a-Set 1) as previously described^40^. We performed 3′RACE on RNA derived from our Skov-3 test cell line and we chose primers that gave us one distinct PCR product (Supplementary Fig. 2). However, when we used the same primers for 3′RACE using RNA derived from HGSOC cell lines, Caov-3 and Ovcar-3, we detected several different sized PCR products (Fig. 2a). After gel purification, TOPO cloning, and Sanger sequencing we found that the smallest product with a 3′UTR of ~ 100 bp (Fig. 2a-product X) contained a poly(A) tail as well as the putative non-canonical polyadenylation signal (PAS) GCCTCC or CCTCCC in both cell lines (Fig. 2b)^41^. The poly(A) tails were not present in the medium sized ~ 300 bp 3′UTR (Fig. 2a-product Y) or the ~ 1000 bp long 3′UTR (Fig. 2a-product Z).

**Figure 3:**
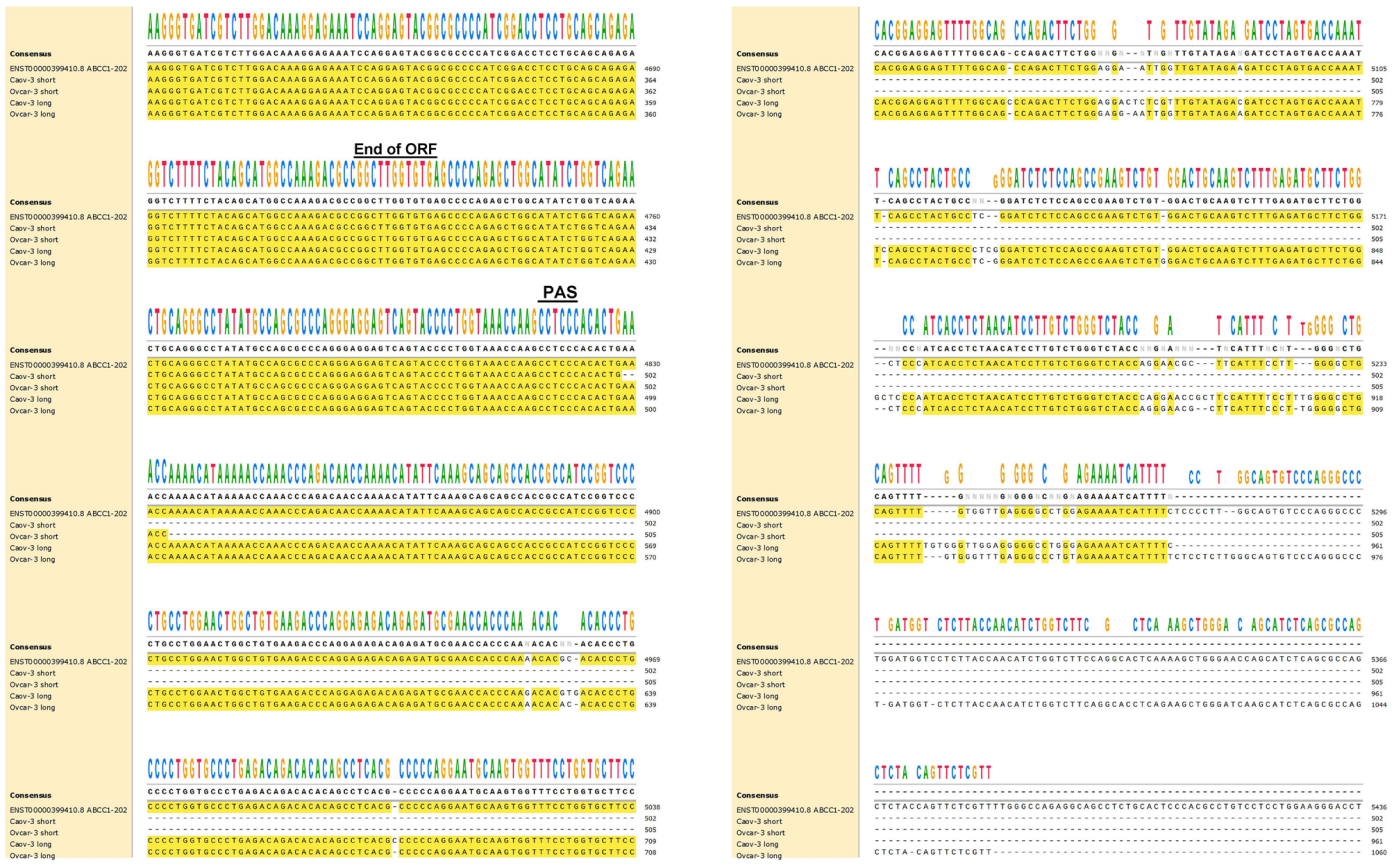
Comparisons of excerpts of *ABCC1* sequences derived from HGSOC cells with the reference sequence transcript from Ensembl genome browser. The consensus sequence is obtained from the reference sequence and the 4 sequences derived from Caov-3 and Ovcar-3 cell lines. The end of the open reading frame (ORF) is shown with the stop codon TGA. The putative polyadenylation signal (PAS) is also depicted. Gaps depict insertions detected in one or more of the HGSOC derived transcripts.

Comparisons of the sequencing products to the NCBI database showed that products X, Y, and Z all mapped to *ABCC1* while the product shown by the asterisk (*) did not. The long and short sequences (X and Z) from both cell lines were compared to each other and aligned to the *ABCC1* cDNA sequence from Ensembl genome browser ENST00000399410.8 ABCC1-202 (matching the NCBI Reference sequence NM_004996.4 formerly NM_004996.3) using the MAFFT SnapGene software (from Insightful Science; available at snapgene.com). Provided is an excerpt showing the sequence alignment in the ORF near the detected PAS (Fig. 3). The complete sequence alignment for the shortest and longest PCR amplicons from 3′RACE is also provided (Supplementary Fig. 2). From our data, of potential significance is the deletion of nucleotide 4338 and/or 4339 from the HGSOC cell lines which occur within the ORF. Interestingly, the region from position 4342 to 4828 (downstream of the stop-codon) contains consensus sequences with no variation between the two HGSOC cell lines. There are several Indels within the 3′UTR we were able to sequence resulting in slight variations between the two cell lines from the reference sequence.

### Determining the link between ferroptosis and *ABCC1* in cisplatin resistant HGSOC

**Figure 4:**
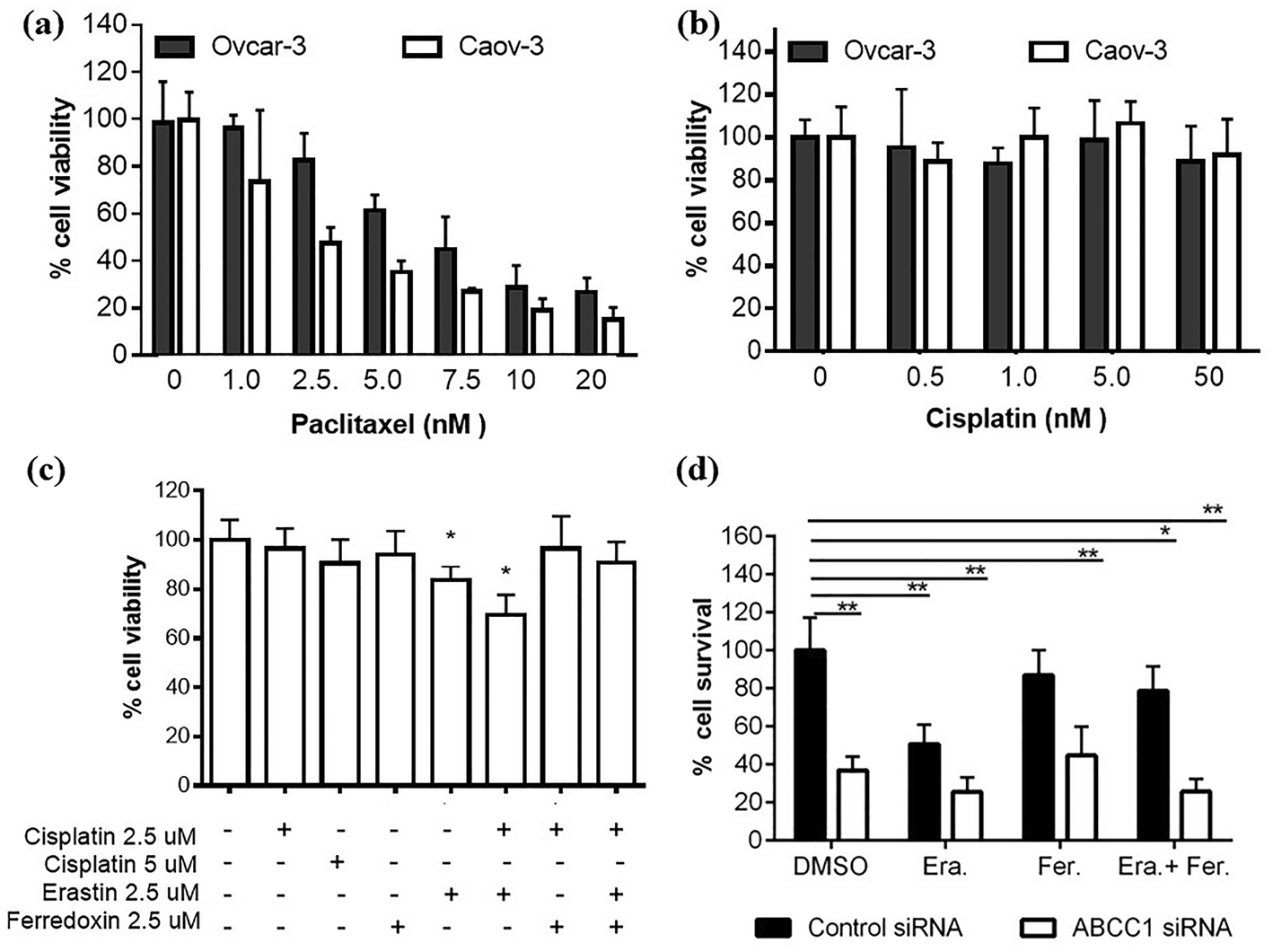
The role of ABCC1 in cisplatin resistance and ferroptosis in ovarian cancer cells. MTT cell viability assay results after treatment of Caov-3 and Ovcar-3 ovarian cancer cells with increasing concentrations of paclitaxel (**a**) or cisplatin (**b**). (**c**) Cell viability assay of the Ovcar-3 cell line after treatment with cisplatin, erastin and ferredoxin. (**d**) Cell viability assay results of Ovcar-3 cells after transfection with control siRNA or siRNA targeting ABCC1 and treatment with erastin (2.5 uM) and ferredoxin (2.5 uM). All results are representative of experiments performed at least twice (n > 4, mean ± s.d., *p* < 0.05) and are normalized to the DMSO delivery vehicle treated control where *p < 0.05, **p < 0.01.

The standard of care for patients with primary ovarian cancer over the past 20 years involves combination chemotherapy treatment, usually with a taxol and platinum compound^42^. The HGSOCs cell lines Caov-3 and Ovcar-3 are reported to be paclitaxel sensitive^43^. We used MTT cell viability assays to verify this information after treating the two different cell lines with increasing concentrations of paclitaxel (Fig. 4a). Both cell lines were sensitive to paclitaxel in the nanomolar range. To determine their sensitivities to cisplatin, the cells were treated with increasing concentrations of cisplatin and MTT assays were then carried out. Consistent with what has been reported, both cell lines were cisplatin resistant (Fig. 4b). Treatment of the Ovcar-3 cell line with up to 5uM of cisplatin did not result in a significant decrease (*p* = 0.14) in cell viability (Fig. 4c). Treatment of cells with 2.5uM of erastin, which induces ferroptosis resulted in a statistically significant decrease in cell viability (*p* = 0.015) and treatment with a combination of cisplatin and erastin resulted in an even greater decrease in cell viability (*p* = 0.0097) (Fig. 4c). The addition of the ferroptosis inhibitor ferredoxin was able to abrogate erastin induced sensitization to cisplatin.

Since the use of erastin was shown to enhance docetaxel-induced apoptosis and increase cell cycle arrest in the cells that overexpressed *ABCB1*^34^, we wanted to determine the impact of using erastin and knocking down *ABCC1* on cisplatin resistant Ovcar-3 cells. Depletion of *ABCC1* using siRNA resulted in a significant decrease in cell viability (*p* = 0.000023) when compared to using control siRNA (Fig. 4d). When compared to the control siRNA, DMSO delivery vehicle treated cells, treatment with erastin resulted in a significant decrease in cell viability for both the control siRNA transfected (*p* = 0.000216) and *ABCC1* depleted (*p* = 0.0000074) cells. Treatment of *ABCC1* siRNA transfected cells with erastin resulted in a significant decrease in cell viability when compared to control siRNA transfected cells treated with erastin (*p* = 0.0028). The ferroptosis inhibitor ferredoxin restored cell viability in erastin treated cells in the control siRNA transfected cells (*p* = 0.015) but not in the *ABCC1* siRNA transfected cells (*p* = 0.93).

### Effect of miRNAs on the ABCC1 3′UTR

**Figure 5:**
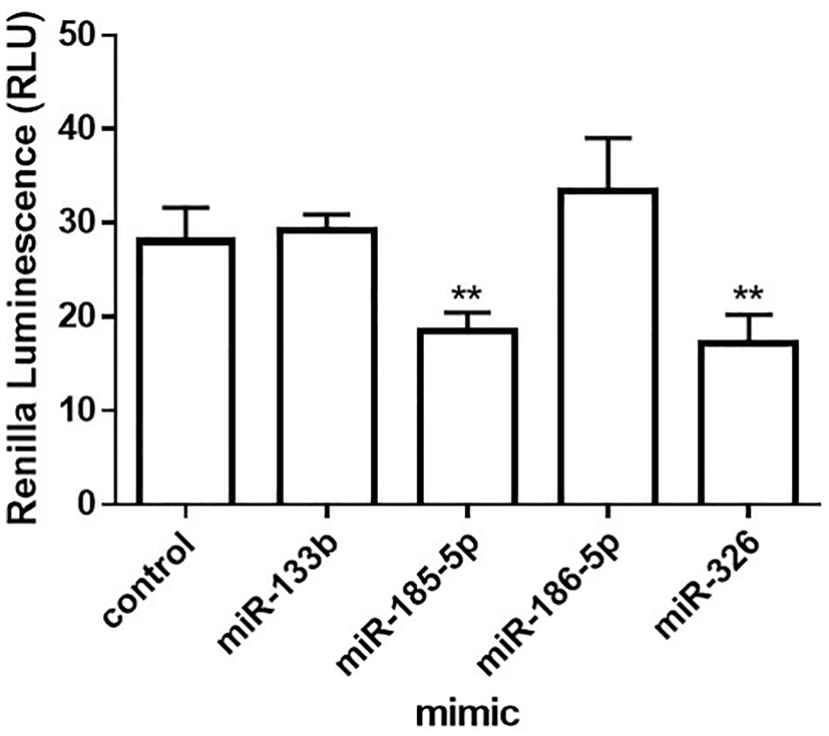
Impact of miRNAs on ABCC1 3′UTR. Graph showing relative luciferase activity of Renilla luciferase normalized to Firefly luciferase in Ovcar-3 cells that were transfected with psicheck2 dual luciferase plasmid containing the full length 3′UTR. This was followed by transfection with different miRNAs mimics and a control. Data are shown as mean and s.d. (n = 3, **p < 0.01).

To determine the impact of different miRNAs on the 3′UTR, the full length 3′UTR of *ABCC1* was cloned into the psiCHECK 2 vector. After transfection with different miRNAs mimics, we found that there was a decrease in luciferase activity after transfection with hsa-miR-185-5p (*p* = 0.0034) and hsa-miR-326 (*p* = 0.0036) (Fig. 5). No significant changes were detected after transfection with the hsa-miR133b and hsa-miR-186-5p.

## Discussion

Alternative polyadenylation (APA) is now being recognized as a major contributor to transcriptome diversity in a similar fashion to mRNAs arising from alternative transcriptome start sites (65%) and alternative splicing (94%)^11–13^. APA that occurs within the same terminal exon (3′UTR-APA), is a form of post-transcriptional regulation that gives rise to mRNAs with different sized 3′UTRs that contain different sequence elements. The 3′UTRs function as loading docks for regulatory elements including RNA binding proteins and miRNAs^44,45^. Shortening of the 3′UTR through 3′UTR-APA would thus eliminate regulation by these factors. The role of 3′UTR-APA in the expression of *ABCC1* in HGSOC is not well understood. Our findings showed that *ABCC1* mRNA levels were similar in Caov-3 cells and the control hFTSEC cell line. Transcripts containing full length 3′UTRs were expressed at the same level as those containing short 3′UTRs. Hence, there are no differences in 3′UTR-APA in the two cell lines with no preference for generating the shorter 3′UTR. However, the protein levels in the Ovcar-3 cell line are higher than those in the hFTSEC cell line. This suggests that the mRNAs in Ovcar-3 are translated at a higher level than those in hFTSECs. This could be due to loss of miRNAs that bind and regulate the stability of the mRNAs in the Ovcar-3 cell line. Interestingly, the Ovcar-3 cell line expresses almost four-fold as much mRNA with the truncated 3′UTR compared to both cell lines, with little expression of the mRNAs with full length 3′UTRs. In this cell line, 3′UTR-APA seems to play a major role in higher expression of *ABCC1* mRNA. The increased levels of Ovcar-3 mRNA also correlate with a three-fold increase in protein levels when compared to the Caov-3 cell line and a sixfold increase in protein expression when compared to the control hFTSEC cell line. Shortening of the 3′UTR through 3′UTR-APA could result in miRNA evasion in the Ovcar-3 cell line allowing for increased translation. This same mechanism linking 3′UTR shortening, and increased protein expression has been reported for many oncogenes^46,47^. Shortening of the 3′UTR of the *ABCC2* drug transporter*,* resulted in loss of miR-329 target sites, and resulted in increased mRNA and protein expression levels^41^. In ovarian cancer, the oncogene survivin also undergoes 3′-UTR shortening, allowing it to escape miRNA regulation^48^. However, increased transcription of *ABCC1* in Ovcar-3 cisplatin resistant cells cannot be fully ruled out. Genome sequencing showed that in 8% of HGSOC recurrent samples, the 5′UTR of *ABCB1* is fused to an upstream promoter which can result in increased transcription^49^. Although the same may be occurring for *ABCC1*, the resulting transcripts would still be subject to miRNA regulation unless they use 3′UTR-APA to generate transcripts with short 3′UTRs to evade miRNAs. MiR-133a and miR-133b were shown to target the 3′UTR of *ABCC1*^50,51^. Other miRNAs that have been reported to target the *ABCC1* 3′UTR are miR-326, miR-503, miR-210-3p, miR-1291^51–54^. Our results show that miR-326 can target the *ABCC1* 3′UTR confirming previous findings. However, miR-133b had no effect on the 3′UTR of *ABCC1*. This could be because we are using a different cell line where additional miR-133b may have other targets which are preferentially bound instead. We also found that addition of the miR-185-5p mimic results in decreased luciferase activity which agrees with previous findings in a study done in lung cancer^55^. Thus, miR-185-5p can regulate the *ABCC1* 3′UTR. Some studies suggest that miR-185 may play a role in cisplatin sensitivity in cancer^55,56^. Since the target site for miR-185-5p is within the longer 3′UTR but is not found within the shorter 3′UTR of *ABCC1*^41^, 3'UTR-APA and thus shortening of the 3′UTR may eliminate regulation by this and other miRNAs.

To generate 3′UTRs of different lengths, 3′end formation occurs ~ 15 nucleotides downstream of alternative polyadenylation signals (PASs). In about 85% of the annotated genome, the PAS consists of the hexameric A(A/U)UAAA sequence^14^. However, other noncanonical PASs can be used in 3′end formation. An early study showed that most non-canonical PASs digress from the aforementioned hexamers by one or more nucleotides with a consensus sequence of NNUANA, where N is any nucleotide^57^. A study by Bruhn et al. suggested that either GCCUCC or CCUCCC (which starts one nucleotide downstream) is the putative PAS’s for the *ABCC1* transcript with the shortest 3′UTR^41^. Our studies also identified the same potential atypical PASs within ~ 10 nucleotides of the polyA tail, in the mRNAs with shortest 3′UTRs (~ 100 bp) in HGSOC. However, since these sequences are atypical and do not fall within the parameters of the consensus sequence (NNUANA), other sequences could also potentially be the PASs signal used since they fall within the 10–30 nucleotides range upstream of the polyA tail (cleavage site). As is the case for other pre-mRNAs, RNA binding proteins which recognize and bind sequences found in *ABCC1,* e.g. UGUA as well as other sequences upstream and downstream of the PAS, may drive 3′end cleavage and polyadenylation of these atypical PASs (as reviewed^58^). The PAS that is used to generate the full length *ABCC1* 3′UTR has more recently been proposed to be AAUAUA which differs by only one nucleotide from the canonical sequence AAUAAA (from the NCBI AceView Database aAug10 to eAug10 with about 39 supporting accessions). Our results also show that there are several nucleotide insertions and deletions within the segment of the longer *ABCC1* 3′UTRs we obtained from the two cell lines, which result in differences from the annotated sequence. These differences may be tissue specific, and their impact remains undetermined.

Over the years, the standard treatment of newly diagnosed epithelial ovarian cancer may include surgical debulking and chemotherapy with a platinum-based (usually carboplatin or cisplatin) chemotherapeutic agent alone or in combination with paclitaxel depending on the tumor stage and other patient-specific factors^59,60^. A major limitation with the platinum based agents is intrinsic and acquired resistance which severely limit their clinical efficacy^61^. Hence, there is a lot of interest in targeted therapies and other treatment approaches based on the tumor’s vulnerabilities to treat epithelial ovarian cancer. Some previous findings suggest that epithelial ovarian cancer may be susceptible to a form of cell death known as ferroptosis^62,63^. In our studies we found that cisplatin resistant HGSOC cells were sensitive to the ferroptosis induced by the small molecule erastin. Furthermore, addition of cisplatin to erastin resulted in a greater decrease in cell viability which was abrogated by treatment with ferredoxin, an inhibitor of ferroptosis. Hence, ferroptosis inducers can potentially be used to sensitize drug resistant tumors to chemotherapy. In a study using the ovarian cancer cell line A2780, cells treated with erastin together with docetaxel had increased apoptosis in cells that overexpressed *ABCB1*^34^. It is however to be noted that the A2780 cell line does not resemble HGSOC tumors^1,35^. To determine whether *ABCC1* was involved in erastin induced ferroptosis in HGSOC, we knocked down *ABCC1* in the Ovcar-3 cell line. Interestingly, *ABCC1* depletion by itself reduced cell viability of HGSOC cells. Two studies suggest that to some extent, there is a correlation between levels of *ABCC1* and tumor grade in ovarian cancer^64,65^. These findings agree with previous suggestions that inhibiting *ABCC1* activity or decreasing its expression can have clinical benefits for a subset of cancer patients^66^. However, ferredoxin was unable to rescue erastin induced ferroptosis after *ABCC1* knockdown suggesting that *ABCC1* is not involved in ferroptosis for HGSOC. The involvement of other ABC transporters in ferroptosis of HGSOC cannot be ruled out. Epithelial ovarian cancer tissues have been shown to express other ABC transporters including, *ABCC3* (MRP3), *ABCC4* (MRP4), *ABCB1* as well as other membrane transporters^65,67,68^. Mechanistic insight would be gained from developing comparable cell lines or xenografts which express known levels of each of the transporters. Furthermore, since cisplatin is largely not regarded as a substrate for *ABCC1*, *ABCC1* is not likely involved in the sensitization to cisplatin after erastin treatment^61^. Our studies suggest that in addition to molecularly targeted therapies, understanding and targeting chemotherapy cancer drug resistance mechanisms should be strongly considered in treating patients with HGSOC. Based on the molecular profile of HGSOC and its potential link to ferroptosis, further investigation into inducing ferroptosis as a therapeutic approach in drug resistant tumors is warranted.

## Conclusions

In HGSOC, 3′UTR-APA plays a critical role as a post-transcriptional regulator of *ABCC1* expression. The *ABCC1* mRNAs that are generated through 3′UTR-APA have different 3′UTR lengths. Higher levels of *ABCC1* transcripts with the short 3′UTRs result in increased protein expression. Shortening of the 3′UTR allows the transcript to escape regulation by miRNAs that bind sequence elements located within the longer 3′UTR. While *ABCC1* does not play a role in inducing regulated cell death through ferroptosis in cisplatin drug resistant HGSOC, depletion of *ABCC1* results in decreased cell viability. In these cisplatin resistant cells, inducing ferroptosis using erastin resulted in decreased cell viability. Hence inducing ferroptosis should be considered as a clinically viable therapeutic option to sensitize cells to cisplatin in drug resistant cells HGSOC.

## Methods

### Cell lines and cell culture conditions

Caov-3, Ovcar-3 and Skov-3 ovarian cancer cell lines were purchased from the America Type Culture Collection (ATCC). Cells were cultured and maintained in DMEM media with Glutamax™ (ThermoFisher Scientific). Human fallopian tube secretory epithelial cells (hFTSECs) were purchased from Applied Biological Materials (British Columbia) and were cultured in DMEM/F12 (ThermoFisher Scientific). All culture media were supplemented with 1% penicillin/streptomycin and 10% heat inactivated fetal bovine serum (both from ThermoFisher Scientific). Cell lines were maintained in a humidified incubator (5% CO_2_) at 37 °C.

### RNA extraction and reverse transcription

Cells were plated in six-well plates and total RNA was extracted using Trizol (ThermoFisher Scientific). Total RNA (2 μg) was reverse transcribed to cDNA using the RevertAid RT kit (ThermoFisher Scientific) as per manufacture’s protocol. Random primers were used for reverse transcription for PCR and qRT-PCR experiments.

### PCR

*ABCC1* specific primers spanning different regions (Fig. 1a) were generated using Primer-BLAST on the *ABCC1* cDNA sequence from Ensembl genome browser ENST00000399410.8 *ABCC1-202* (matching the NCBI Reference sequence NM_004996.4 formerly NM_004996.3). Different combinations of primers were used for PCR using Extender PCR to Gel Mastermix (Amresco]). The amplicons were then run on a 2% agarose gel stained with ethidium bromide. PCR was done using the Mastercycler PCR System (Eppendorf).

### qRT-PCR

A set of primers in the *ABCC1* ORF and in the 3′UTR region (Fig. 1a) were used for qRT-PCR with GAPDH as a loading control. KAPA SYBRFAST (Roche) was used for the qRT-PCR reaction. Normalization was done to GAPDH using the 2^–∆∆Ct^ method. *ABCC1* Primers used for the ORF were Forward Primer 5′-GCTCGTCTTGTCCTGTTTCT-3′ and Reverse Primer 5′-GCATTCCTTCTTCCAGTTCTTTAC-3′. The *ABCC1* 3′UTR primers were Forward 5′-GCAGTGTTGGTTGCTTACAG-3′ and Reverse 5′-GGATGCCAAGGGAGAGAATTA-3′. The qRT-PCR was done using the CFX96 real time system (BioRad).

### 3′RACE and sequencing

For 3′RACE the total RNA was DNase treated (Promega, WI) and oligo(dT_25_)T7 primers were used for reverse transcription. The 3′RACE reactions were performed using Phusion master Mix (ThermoFisher Scientific) and reverse primers as previously described^40^. To simultaneously detect all forms of *ABCC1* 3′UTRs, we used the nested primers within the open reading frame of *ABCC1*. The sequence of the forward primer used for the first round of PCR was 5′-GACCTCCGCTTCAAGATCAC-3′ and for the second PCR was 5′-GAATGAACCTGGACCCATTCA-3′. After gel purification using the gel extraction wizard SV gel and PCR cleanup system (Promega), the second round PCR products were cloned using Zero Blunt TOPO PCR Cloning Kit for Sequencing (Life Technologies) and verified by Sanger sequencing (Lonestar Labs).

### Western blotting

Cells were plated in 6-well plates and cultured cells were lysed with M-PER mammalian protein extraction reagent (ThermoFisher Scientific) supplemented with Halt protease inhibitor cocktail (Life Technologies). The cell lysate was loaded onto an SDS–polyacrylamide gel. After electrophoresis, the separated proteins were wet transferred to a PVDF membrane (BioRad). The membrane was blocked in 5% nonfat milk in phosphate buffered saline with 0.01% Tween(PBS-T), and incubated with the *ABCC1*(*MRP1*) primary antibody (1:1000 dilution-Bethyl Lab A304-419-A) and the GAPDH antibody (1:5,000 dilution- Abcam ab128915) overnight at 4 °C. Following incubation with the primary antibody, the membrane was washed three times with PBS-T. The membrane was incubated with the goat-anti-rabbit IgG Superclonal Alexa Fluor®680 secondary antibody (ThermoFisher Scientific) at 1:5000 dilution for one hour at room temperature. After washing three times with PBS-T, the bands on the membrane were visualized using FluorChem Q MultiImager (Alpha Innotech). The bands were quantified using Image J and levels of *ABCC1* were normalized to levels of *GAPDH*.

### MTT assays

For MTT assays, cells (7500 per well) were seeded in 96-well plates. To determine sensitivity to chemotherapy, after 24 h, cells were treated with different doses of cisplatin (Millipore Sigma) or paclitaxel (ThermoFisher Scientific) for 72 h. To determine the impact of erastin, 24 h after culturing in 96 well plates, cells were treated with cisplatin, erastin (Selleck Chemicals, TX) and ferredoxin(Selleck Chemicals, TX)) either alone or in combination for 72 h. For the RNAi experiment, cells were reverse transfected with negative control siRNA (SIC002) or *ABCC1* siRNA (SASI_Hs01_00155530) from SigmaAldrich using Lipofectamine 2000 (ThermoFisher Scientific) as per manufacturer’s protocol. Twenty-four hours post-transfection, cells were treated with erastin and/or ferredoxin for 48 h. For each experiment, the delivery vehicle DMSO was used as the control treatment. The MTT assay was performed after the appropriate treatment as per the manufacturer’s protocol (ATCC) with a few modifications. Briefly, 10 μl of MTT was added to each well and incubated in a 5% CO_2_ incubator at 37 °C for 2–4 h. Then 100 μl of detergent was added and the plate was incubated at room temperature in a moist, dark chamber and absorbance was measured at 570 nm using the Biotek Synergy two plate reader Gen5 software (BioTek Instruments).

### Cloning and luciferase assays

The full length 3′UTR of *ABCC1* was cloned into the psiCHECK-2 plasmid (Promega) between the Not1 and Xho1 restriction enzyme sites adjacent to the human Renilla luciferase sequence (GenScript). We reverse transfected 1ug of plasmid per well in a 12 well plate with Ovcar-3 cells using Lipofectamine 2000 (ThermoFisher Scientific). After 24 h we transfected cells with the miRNA mimic negative control (4,464,058), hsa-miR-326 mimic (MC10686), hsa-miR-133b mimic (MC10029), hsa-miR-186-5p mimic (MC11753), hsa-miR-185-5p mimic (MC12486) from Life Technologies. After 48 h we collected used the dual-luciferase reporter assay kit (Promega) to collect the cell lysate and measure firefly luminescence followed by Renilla luminescence as per manufacture’s protocol.

### Statistical analysis

The statistical analysis was carried out using GraphPad Prism 6 software. ANOVA and/or the student’s t-test was used to determine statistical significance.

### Supplementary Information

Supplementary Figures.

## Data Availability

The sequencing data supporting the findings of this study was deposited with NCBI-GenBank (accession numbers OQ859720, OQ859721, OQ859722 and OQ859723).

## Abbreviations

ABC: ATP-binding cassette transporter
ABCC1: ATP binding cassette subfamily C member 1
APA: Alternative polyadenylation
HGSOC: High grade serous ovarian cancer
hFTSECs: Human fallopian tube fimbria secretory epithelial cells
PCR: Polymerase chain reaction
qRT-PCR: Real time quantitative reverse transcription polymerase chain reaction
3′UTR: 3′Untranslated region
3′RACE: 3′ Rapid amplification of cDNA ends
