# Supplementary material for: The role and impact of alternative polyadenylation and miRNA regulation on the expression of the multidrug resistance-associated protein 1 (MRP-1/ABCC1) in epithelial ovarian cancer
Source: Sci Rep. 2023 Oct 14;13:17476. doi: 10.1038/s41598-023-44548-y (PMC10576765; doi:10.1038/s41598-023-44548-y)
Supplement: Supplementary file 1 — Supplementary Figures. [file 41598_2023_44548_MOESM1_ESM.pdf]

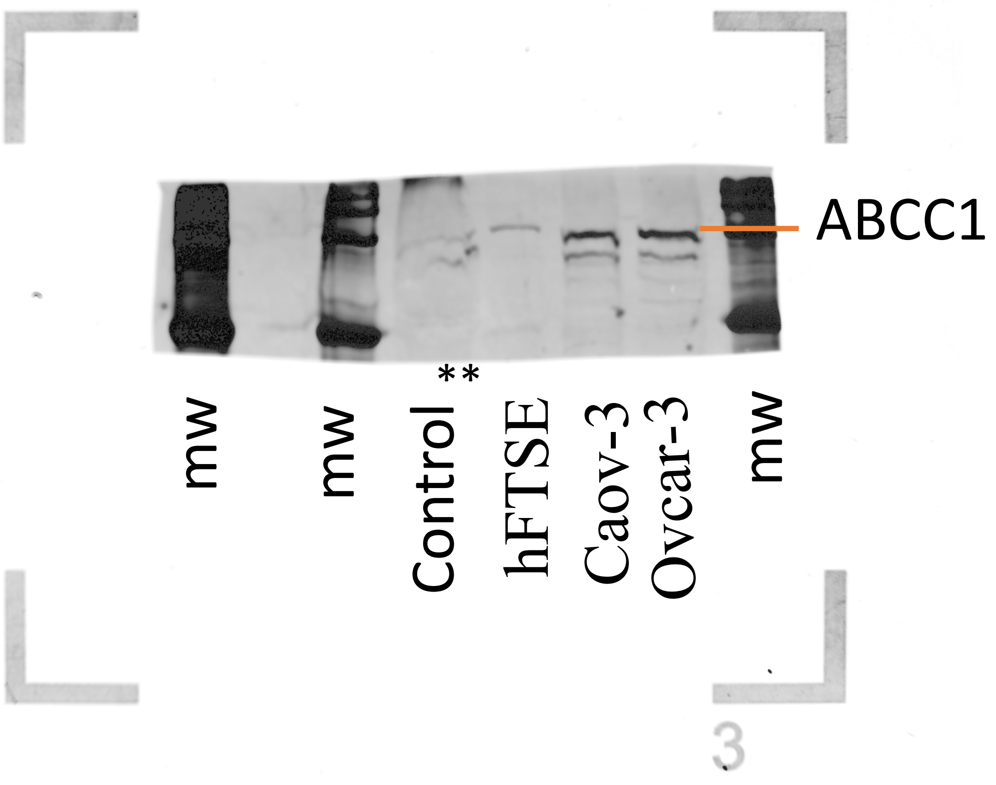

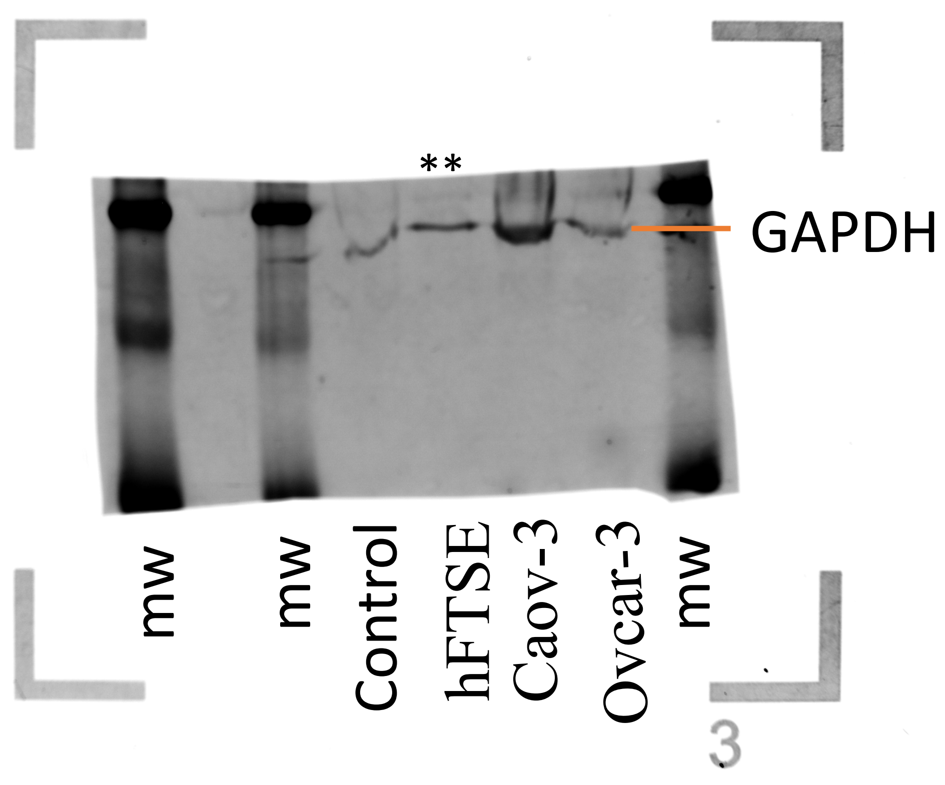

Supplemental Figure 1A. Western Blot for ABCC1. Shown is the full membrane from the Imager that was cut to probe for *ABCC1* and GAPDH and provide data for Figure 1C. The molecular weight (mw) markers are labeled. Please note that the cut membrane fits together on the edges with the **.

	Densitometry readings using Image J		
**cell line**	**ABCC1 area**	**GAPDH area**	**divided by GAPDH**
hFTSECs	2152.569	10496.681	0.2
Caov-3	15770.433	24207.342	0.7
Ovcar-3	16633.513	9877.359	1.7

Supplemental Figure 1B. Shown are the densitometry readings obtained using Image J used on western blots from Supplementary Figure 1A and this shows the area under each band and the final value when normalized to GAPDH.

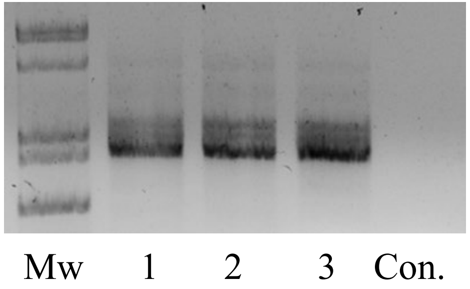

Supplementary Figure 2. Primers for ABCC1 3'RACE. An Ethidium bromide stained agarose gel of 3'RACE products using primers designed to detect 3'UTRs of ABCC1 in RNA derived from three different SKOV3 cell line extracts and a control (Con) sample with no RNA.

```
Consensus                    ----------------------------------------------------------------------
ENST00000399410.8 ABCC1-202  GCCGCCGCCGCCGCCGCCAGCGCTAGCGCCAGCAGCCGGGCCCGATCACCCGCCGCCCGGTGCCCGCCGC  70
Caov-3 short                 ----------------------------------------------------------------------
Ovcar-3 short                ----------------------------------------------------------------------
Caov-3 long                  ----------------------------------------------------------------------
Ovcar-3 long                 ----------------------------------------------------------------------

Consensus                    ----------------------------------------------------------------------
ENST00000399410.8 ABCC1-202  CGCCCGCGCCAGCAACCGGGCCCGATCACCCGCCGCCCGGTGCCCGCCGCCGCCCGCGCCACCGGCATGG  140
Caov-3 short                 ----------------------------------------------------------------------
Ovcar-3 short                ----------------------------------------------------------------------
Caov-3 long                  ----------------------------------------------------------------------
Ovcar-3 long                 ----------------------------------------------------------------------

Consensus                    ----------------------------------------------------------------------
ENST00000399410.8 ABCC1-202  CGCTCCGGGGCTTCTGCAGCGCCGATGGCTCCGACCCGCTCTGGGACTGGAATGTCACGTGGAATACCAG  210
Caov-3 short                 ----------------------------------------------------------------------
Ovcar-3 short                ----------------------------------------------------------------------
Caov-3 long                  ----------------------------------------------------------------------
Ovcar-3 long                 ----------------------------------------------------------------------

Consensus                    ----------------------------------------------------------------------
ENST00000399410.8 ABCC1-202  CAACCCCGACTTCACCAAGTGCTTTCAGAACACGGTCCTCGTGTGGGTGCCTTGTTTTTACCTCTGGGCC  280
Caov-3 short                 ----------------------------------------------------------------------
Ovcar-3 short                ----------------------------------------------------------------------
Caov-3 long                  ----------------------------------------------------------------------
Ovcar-3 long                 ----------------------------------------------------------------------

Consensus                    ----------------------------------------------------------------------
ENST00000399410.8 ABCC1-202  TGTTTCCCCTTCTACTTCCTCTATCTCTCCCGACATGACCGAGGCTACATTCAGATGACACCTCTCAACA  350
Caov-3 short                 ----------------------------------------------------------------------
Ovcar-3 short                ----------------------------------------------------------------------
Caov-3 long                  ----------------------------------------------------------------------
Ovcar-3 long                 ----------------------------------------------------------------------

Consensus                    ----------------------------------------------------------------------
ENST00000399410.8 ABCC1-202  AAACCAAAACTGCCTTGGGATTTTTGCTGTGGATCGTCTGCTGGGCAGACCTCTTCTACTCTTTCTGGGA  420
Caov-3 short                 ----------------------------------------------------------------------
Ovcar-3 short                ----------------------------------------------------------------------
Caov-3 long                  ----------------------------------------------------------------------
Ovcar-3 long                 ----------------------------------------------------------------------
```

```
Consensus                     ----------------------------------------------------------------------
ENST00000399410.8 ABCC1-202   AAGAAGTCGGGGCATATTCCTGGCCCCAGTGTTTCTGGTCAGCCCAACTCTCTTGGGCATCACCATGCTG  490
Caov-3 short                  ----------------------------------------------------------------------
Ovcar-3 short                 ----------------------------------------------------------------------
Caov-3 long                   ----------------------------------------------------------------------
Ovcar-3 long                  ----------------------------------------------------------------------

Consensus                     ----------------------------------------------------------------------
ENST00000399410.8 ABCC1-202   CTTGCTACCTTTTTAATTCAGCTGGAGAGGAGGAAGGGAGTTCAGTCTTCAGGGATCATGCTCACTTTCT  560
Caov-3 short                  ----------------------------------------------------------------------
Ovcar-3 short                 ----------------------------------------------------------------------
Caov-3 long                   ----------------------------------------------------------------------
Ovcar-3 long                  ----------------------------------------------------------------------

Consensus                     ----------------------------------------------------------------------
ENST00000399410.8 ABCC1-202   GGCTGGTAGCCCTAGTGTGTGCCCTAGCCATCCTGAGATCCAAAATTATGACAGCCTTAAAAGAGGATGC  630
Caov-3 short                  ----------------------------------------------------------------------
Ovcar-3 short                 ----------------------------------------------------------------------
Caov-3 long                   ----------------------------------------------------------------------
Ovcar-3 long                  ----------------------------------------------------------------------

Consensus                     ----------------------------------------------------------------------
ENST00000399410.8 ABCC1-202   CCAGGTGGACCTGTTTCGTGACATCACTTTCTACGTCTACTTTTCCCTCTTACTCATTCAGCTCGTCTTG  700
Caov-3 short                  ----------------------------------------------------------------------
Ovcar-3 short                 ----------------------------------------------------------------------
Caov-3 long                   ----------------------------------------------------------------------
Ovcar-3 long                  ----------------------------------------------------------------------

Consensus                     ----------------------------------------------------------------------
ENST00000399410.8 ABCC1-202   TCCTGTTTCTCAGATCGCTCACCCCTGTTCTCGGAAACCATCCACGACCCTAATCCCTGCCCAGAGTCCA  770
Caov-3 short                  ----------------------------------------------------------------------
Ovcar-3 short                 ----------------------------------------------------------------------
Caov-3 long                   ----------------------------------------------------------------------
Ovcar-3 long                  ----------------------------------------------------------------------

Consensus                     ----------------------------------------------------------------------
ENST00000399410.8 ABCC1-202   GCGCTTCCTTCCTGTCGAGGATCACCTTCTGGTGGATCACAGGGTTGATTGTCCGGGGCTACCGCCAGCC  840
Caov-3 short                  ----------------------------------------------------------------------
Ovcar-3 short                 ----------------------------------------------------------------------
Caov-3 long                   ----------------------------------------------------------------------
Ovcar-3 long                  ----------------------------------------------------------------------
```

**Consensus**	--	
ENST00000399410.8 ABCC1-202	CCTGGAGGGCAGTGACCTCTGGTCCTTAAACAAGGAGGACACGTCGGAACAAGTCGTGCCTGTTTTGGTA	910
Caov-3 short	--	
Ovcar-3 short	--	
Caov-3 long	--	
Ovcar-3 long	--	

**Consensus**	--	
ENST00000399410.8 ABCC1-202	AAGAACTGGAAGAAGGAATGCGCCAAGACTAGGAAGCAGCCGGTGAAGGTTGTGTACTCCTCCAAGGATC	980
Caov-3 short	--	
Ovcar-3 short	--	
Caov-3 long	--	
Ovcar-3 long	--	

**Consensus**	--	
ENST00000399410.8 ABCC1-202	CTGCCCAGCCGAAAGAGAGTTCCAAGGTGGATGCGAATGAGGAGGTGGAGGCTTTGATCGTCAAGTCCCC	1050
Caov-3 short	--	
Ovcar-3 short	--	
Caov-3 long	--	
Ovcar-3 long	--	

**Consensus**	--	
ENST00000399410.8 ABCC1-202	ACAGAAGGAGTGGAACCCCTCTCTGTTTAAGGTGTTATACAAGACCTTTGGGCCCTACTTCCTCATGAGC	1120
Caov-3 short	--	
Ovcar-3 short	--	
Caov-3 long	--	
Ovcar-3 long	--	

**Consensus**	--	
ENST00000399410.8 ABCC1-202	TTCTTCTTCAAGGCCATCCACGACCTGATGATGTTTTCCGGGCCGCAGATCTTAAAGTTGCTCATCAAGT	1190
Caov-3 short	--	
Ovcar-3 short	--	
Caov-3 long	--	
Ovcar-3 long	--	

**Consensus**	--	
ENST00000399410.8 ABCC1-202	TCGTGAATGACACGAAGGCCCCAGACTGGCAGGGCTACTTCTACACCGTGCTGCTGTTTGTCACTGCCTG	1260
Caov-3 short	--	
Ovcar-3 short	--	
Caov-3 long	--	
Ovcar-3 long	--	

**Consensus**	--	
ENST00000399410.8 ABCC1-202	CCTGCAGACCCTCGTGCTGCACCAGTACTTCCACATCTGCTTCGTCAGTGGCATGAGGATCAAGACCGCT	1330
Caov-3 short	--	
Ovcar-3 short	--	
Caov-3 long	--	
Ovcar-3 long	--	

**Consensus**	--	
ENST00000399410.8 ABCC1-202	GTCATTGGGGCTGTCTATCGGAAGGCCCTGGTGATCACCAATTCAGCCAGAAAATCCTCCACGGTCGGGG	1400
Caov-3 short	--	
Ovcar-3 short	--	
Caov-3 long	--	
Ovcar-3 long	--	

**Consensus**	--	
ENST00000399410.8 ABCC1-202	AGATTGTCAACCTCATGTCTGTGGACGCTCAGAGGTTCATGGACTTGGCCACGTACATTAACATGATCTG	1470
Caov-3 short	--	
Ovcar-3 short	--	
Caov-3 long	--	
Ovcar-3 long	--	

**Consensus**	--	
ENST00000399410.8 ABCC1-202	GTCAGCCCCCCTGCAAGTCATCCTTGCTCTCTACCTCCTGTGGCTGAATCTGGGCCCTTCCGTCCTGGCT	1540
Caov-3 short	--	
Ovcar-3 short	--	
Caov-3 long	--	
Ovcar-3 long	--	

**Consensus**	--	
ENST00000399410.8 ABCC1-202	GGAGTGGCGGTGATGGTCCTCATGGTGCCCGTCAATGCTGTGATGGCGATGAAGACCAAGACGTATCAGG	1610
Caov-3 short	--	
Ovcar-3 short	--	
Caov-3 long	--	
Ovcar-3 long	--	

**Consensus**	--	
ENST00000399410.8 ABCC1-202	TGGCCCACATGAAGAGCAAAGACAATCGGATCAAGCTGATGAACGAAATTCTCAATGGGATCAAAGTGCT	1680
Caov-3 short	--	
Ovcar-3 short	--	
Caov-3 long	--	
Ovcar-3 long	--	

```
Consensus                    ----------------------------------------------------------------------
ENST00000399410.8 ABCC1-202  AAAGCTTTATGCCTGGGAGCTGGCATTCAAGGACAAGGTGCTGGCCATCAGGCAGGAGGAGCTGAAGGTG  1750
Caov-3 short                 ----------------------------------------------------------------------
Ovcar-3 short                ----------------------------------------------------------------------
Caov-3 long                  ----------------------------------------------------------------------
Ovcar-3 long                 ----------------------------------------------------------------------
```

```
Consensus                    ----------------------------------------------------------------------
ENST00000399410.8 ABCC1-202  CTGAAGAAGTCTGCCTACCTGTCAGCCGTGGGCACCTTCACCTGGGTCTGCACGCCCTTTCTGGTGGCCT  1820
Caov-3 short                 ----------------------------------------------------------------------
Ovcar-3 short                ----------------------------------------------------------------------
Caov-3 long                  ----------------------------------------------------------------------
Ovcar-3 long                 ----------------------------------------------------------------------
```

```
Consensus                    ----------------------------------------------------------------------
ENST00000399410.8 ABCC1-202  TGTGCACATTTGCCGTCTACGTGACCATTGACGAGAACAACATCCTGGATGCCCAGACAGCCTTCGTGTC  1890
Caov-3 short                 ----------------------------------------------------------------------
Ovcar-3 short                ----------------------------------------------------------------------
Caov-3 long                  ----------------------------------------------------------------------
Ovcar-3 long                 ----------------------------------------------------------------------
```

```
Consensus                    ----------------------------------------------------------------------
ENST00000399410.8 ABCC1-202  TTTGGCCTTGTTCAACATCCTCCGGTTTCCCCTGAACATTCTCCCCATGGTCATCAGCAGCATCGTGCAG  1960
Caov-3 short                 ----------------------------------------------------------------------
Ovcar-3 short                ----------------------------------------------------------------------
Caov-3 long                  ----------------------------------------------------------------------
Ovcar-3 long                 ----------------------------------------------------------------------
```

```
Consensus                    ----------------------------------------------------------------------
ENST00000399410.8 ABCC1-202  GCGAGTGTCTCCCTCAAACGCCTGAGGATCTTTCTCTCCCATGAGGAGCTGGAACCTGACAGCATCGAGC  2030
Caov-3 short                 ----------------------------------------------------------------------
Ovcar-3 short                ----------------------------------------------------------------------
Caov-3 long                  ----------------------------------------------------------------------
Ovcar-3 long                 ----------------------------------------------------------------------
```

```
Consensus                    ----------------------------------------------------------------------
ENST00000399410.8 ABCC1-202  GACGGCCTGTCAAAGACGGCGGGGGCACGAACAGCATCACCGTGAGGAATGCCACATTCACCTGGGCCAG  2100
Caov-3 short                 ----------------------------------------------------------------------
Ovcar-3 short                ----------------------------------------------------------------------
Caov-3 long                  ----------------------------------------------------------------------
Ovcar-3 long                 ----------------------------------------------------------------------
```

```
Consensus                     ----------------------------------------------------------------------
ENST00000399410.8 ABCC1-202   GAGCGACCCTCCCACACTGAATGGCATCACCTTCTCCATCCCCGAAGGTGCTTTGGTGGCCGTGGTGGGC  2170
Caov-3 short                  ----------------------------------------------------------------------
Ovcar-3 short                 ----------------------------------------------------------------------
Caov-3 long                   ----------------------------------------------------------------------
Ovcar-3 long                  ----------------------------------------------------------------------

Consensus                     ----------------------------------------------------------------------
ENST00000399410.8 ABCC1-202   CAGGTGGGCTGCGGAAAGTCGTCCCTGCTCTCAGCCCTCTTGGCTGAGATGGACAAAGTGGAGGGGCACG  2240
Caov-3 short                  ----------------------------------------------------------------------
Ovcar-3 short                 ----------------------------------------------------------------------
Caov-3 long                   ----------------------------------------------------------------------
Ovcar-3 long                  ----------------------------------------------------------------------

Consensus                     ----------------------------------------------------------------------
ENST00000399410.8 ABCC1-202   TGGCTATCAAGGGCTCCGTGGCCTATGTGCCACAGCAGGCCTGGATTCAGAATGATTCTCTCCGAGAAAA  2310
Caov-3 short                  ----------------------------------------------------------------------
Ovcar-3 short                 ----------------------------------------------------------------------
Caov-3 long                   ----------------------------------------------------------------------
Ovcar-3 long                  ----------------------------------------------------------------------

Consensus                     ----------------------------------------------------------------------
ENST00000399410.8 ABCC1-202   CATCCTTTTTGGATGTCAGCTGGAGGAACCATATTACAGGTCCGTGATACAGGCCTGTGCCCTCCTCCCA  2380
Caov-3 short                  ----------------------------------------------------------------------
Ovcar-3 short                 ----------------------------------------------------------------------
Caov-3 long                   ----------------------------------------------------------------------
Ovcar-3 long                  ----------------------------------------------------------------------

Consensus                     ----------------------------------------------------------------------
ENST00000399410.8 ABCC1-202   GACCTGGAAATCCTGCCCAGTGGGGATCGGACAGAGATTGGCGAGAAGGGCGTGAACCTGTCTGGGGGCC  2450
Caov-3 short                  ----------------------------------------------------------------------
Ovcar-3 short                 ----------------------------------------------------------------------
Caov-3 long                   ----------------------------------------------------------------------
Ovcar-3 long                  ----------------------------------------------------------------------

Consensus                     ----------------------------------------------------------------------
ENST00000399410.8 ABCC1-202   AGAAGCAGCGCGTGAGCCTGGCCCGGGCCGTGTACTCCAACGCTGACATTTACCTCTTCGATGATCCCCT  2520
Caov-3 short                  ----------------------------------------------------------------------
Ovcar-3 short                 ----------------------------------------------------------------------
Caov-3 long                   ----------------------------------------------------------------------
Ovcar-3 long                  ----------------------------------------------------------------------
```

**Consensus**	--	
ENST00000399410.8 ABCC1-202	CTCAGCAGTGGATGCCCATGTGGGAAAACACATCTTTGAAAATGTGATTGGCCCCAAGGGGATGCTGAAG	2590
Caov-3 short	--	
Ovcar-3 short	--	
Caov-3 long	--	
Ovcar-3 long	--	

**Consensus**	--	
ENST00000399410.8 ABCC1-202	AACAAGACGCGGATCTTGGTCACGCACAGCATGAGCTACTTGCCGCAGGTGGACGTCATCATCGTCATGA	2660
Caov-3 short	--	
Ovcar-3 short	--	
Caov-3 long	--	
Ovcar-3 long	--	

**Consensus**	--	
ENST00000399410.8 ABCC1-202	GTGGCGGCAAGATCTCTGAGATGGGCTCCTACCAGGAGCTGCTGGCTCGAGACGGCGCCTTCGCTGAGTT	2730
Caov-3 short	--	
Ovcar-3 short	--	
Caov-3 long	--	
Ovcar-3 long	--	

**Consensus**	--	
ENST00000399410.8 ABCC1-202	CCTGCGTACCTATGCCAGCACAGAGCAGGAGCAGGATGCAGAGGAGAACGGGGTCACGGGCGTCAGCGGT	2800
Caov-3 short	--	
Ovcar-3 short	--	
Caov-3 long	--	
Ovcar-3 long	--	

**Consensus**	--	
ENST00000399410.8 ABCC1-202	CCAGGGAAGGAAGCAAAGCAAATGGAGAATGGCATGCTGGTGACGGACAGTGCAGGGAAGCAACTGCAGA	2870
Caov-3 short	--	
Ovcar-3 short	--	
Caov-3 long	--	
Ovcar-3 long	--	

**Consensus**	--	
ENST00000399410.8 ABCC1-202	GACAGCTCAGCAGCTCCTCCTCCTATAGTGGGGACATCAGCAGGCACCACAACAGCACCGCAGAACTGCA	2940
Caov-3 short	--	
Ovcar-3 short	--	
Caov-3 long	--	
Ovcar-3 long	--	

```
Consensus                      ----------------------------------------------------------------------
ENST00000399410.8 ABCC1-202    GAAAGCTGAGGCCAAGAAGGAGGAGACCTGGAAGCTGATGGAGGCTGACAAGGCGCAGACAGGGCAGGTC  3010
Caov-3 short                   ----------------------------------------------------------------------
Ovcar-3 short                  ----------------------------------------------------------------------
Caov-3 long                    ----------------------------------------------------------------------
Ovcar-3 long                   ----------------------------------------------------------------------

Consensus                      ----------------------------------------------------------------------
ENST00000399410.8 ABCC1-202    AAGCTTTCCGTGTACTGGGACTACATGAAGGCCATCGGACTCTTCATCTCCTTCCTCAGCATCTTCCTTT  3080
Caov-3 short                   ----------------------------------------------------------------------
Ovcar-3 short                  ----------------------------------------------------------------------
Caov-3 long                    ----------------------------------------------------------------------
Ovcar-3 long                   ----------------------------------------------------------------------

Consensus                      ----------------------------------------------------------------------
ENST00000399410.8 ABCC1-202    TCATGTGTAACCATGTGTCCGCGCTGGCTTCCAACTATTGGCTCAGCCTCTGGACTGATGACCCCATCGT  3150
Caov-3 short                   ----------------------------------------------------------------------
Ovcar-3 short                  ----------------------------------------------------------------------
Caov-3 long                    ----------------------------------------------------------------------
Ovcar-3 long                   ----------------------------------------------------------------------

Consensus                      ----------------------------------------------------------------------
ENST00000399410.8 ABCC1-202    CAACGGGACTCAGGAGCACACGAAAGTCCGGCTGAGCGTCTATGGAGCCCTGGGCATTTCACAAGGGATC  3220
Caov-3 short                   ----------------------------------------------------------------------
Ovcar-3 short                  ----------------------------------------------------------------------
Caov-3 long                    ----------------------------------------------------------------------
Ovcar-3 long                   ----------------------------------------------------------------------

Consensus                      ----------------------------------------------------------------------
ENST00000399410.8 ABCC1-202    GCCGTGTTTGGCTACTCCATGGCCGTGTCCATCGGGGGGATCTTGGCTTCCCGCTGTCTGCACGTGGACC  3290
Caov-3 short                   ----------------------------------------------------------------------
Ovcar-3 short                  ----------------------------------------------------------------------
Caov-3 long                    ----------------------------------------------------------------------
Ovcar-3 long                   ----------------------------------------------------------------------

Consensus                      ----------------------------------------------------------------------
ENST00000399410.8 ABCC1-202    TGCTGCACAGCATCCTGCGGTCACCCATGAGCTTCTTTGAGCGGACCCCCAGTGGGAACCTGGTGAACCG  3360
Caov-3 short                   ----------------------------------------------------------------------
Ovcar-3 short                  ----------------------------------------------------------------------
Caov-3 long                    ----------------------------------------------------------------------
Ovcar-3 long                   ----------------------------------------------------------------------
```

```
Consensus                      ----------------------------------------------------------------------
ENST00000399410.8 ABCC1-202    CTTCTCCAAGGAGCTGGACACAGTGGACTCCATGATCCCGGAGGTCATCAAGATGTTCATGGGCTCCCTG  3430
Caov-3 short                   ----------------------------------------------------------------------
Ovcar-3 short                  ----------------------------------------------------------------------
Caov-3 long                    ----------------------------------------------------------------------
Ovcar-3 long                   ----------------------------------------------------------------------

Consensus                      ----------------------------------------------------------------------
ENST00000399410.8 ABCC1-202    TTCAACGTCATTGGTGCCTGCATCGTTATCCTGCTGGCCACGCCCATCGCCGCCATCATCATCCCGCCCC  3500
Caov-3 short                   ----------------------------------------------------------------------
Ovcar-3 short                  ----------------------------------------------------------------------
Caov-3 long                    ----------------------------------------------------------------------
Ovcar-3 long                   ----------------------------------------------------------------------

Consensus                      ----------------------------------------------------------------------
ENST00000399410.8 ABCC1-202    TTGGCCTCATCTACTTCTTCGTCCAGAGGTTCTACGTGGCTTCCTCCCGGCAGCTGAAGCGCCTCGAGTC  3570
Caov-3 short                   ----------------------------------------------------------------------
Ovcar-3 short                  ----------------------------------------------------------------------
Caov-3 long                    ----------------------------------------------------------------------
Ovcar-3 long                   ----------------------------------------------------------------------

Consensus                      ----------------------------------------------------------------------
ENST00000399410.8 ABCC1-202    GGTCAGCCGCTCCCCGGTCTATTCCCATTTCAACGAGACCTTGCTGGGGGTCAGCGTCATTCGAGCCTTC  3640
Caov-3 short                   ----------------------------------------------------------------------
Ovcar-3 short                  ----------------------------------------------------------------------
Caov-3 long                    ----------------------------------------------------------------------
Ovcar-3 long                   ----------------------------------------------------------------------

Consensus                      ----------------------------------------------------------------------
ENST00000399410.8 ABCC1-202    GAGGAGCAGGAGCGCTTCATCCACCAGAGTGACCTGAAGGTGGACGAGAACCAGAAGGCCTATTACCCCA  3710
Caov-3 short                   ----------------------------------------------------------------------
Ovcar-3 short                  ----------------------------------------------------------------------
Caov-3 long                    ----------------------------------------------------------------------
Ovcar-3 long                   ----------------------------------------------------------------------

Consensus                      ----------------------------------------------------------------------
ENST00000399410.8 ABCC1-202    GCATCGTGGCCAACAGGTGGCTGGCCGTGCGGCTGGAGTGTGTGGGCAACTGCATCGTTCTGTTTGCTGC  3780
Caov-3 short                   ----------------------------------------------------------------------
Ovcar-3 short                  ----------------------------------------------------------------------
Caov-3 long                    ----------------------------------------------------------------------
Ovcar-3 long                   ----------------------------------------------------------------------
```

**Consensus**	--	
ENST00000399410.8 ABCC1-202	CCTGTTTGCGGTGATCTCCAGGCACAGCCTCAGTGCTGGCTTGGTGGGCCTCTCAGTGTCTTACTCATTG	3850
Caov-3 short	--	
Ovcar-3 short	--	
Caov-3 long	--	
Ovcar-3 long	--	
**Consensus**	--	
ENST00000399410.8 ABCC1-202	CAGGTCACCACGTACTTGAACTGGCTGGTTCGGATGTCATCTGAAATGGAAACCAACATCGTGGCCGTGG	3920
Caov-3 short	--	
Ovcar-3 short	--	
Caov-3 long	--	
Ovcar-3 long	--	
**Consensus**	--	
ENST00000399410.8 ABCC1-202	AGAGGCTCAAGGAGTATTCAGAGACTGAGAAGGAGGCGCCCTGGCAAATCCAGGAGACAGCTCCGCCCAG	3990
Caov-3 short	--	
Ovcar-3 short	--	
Caov-3 long	--	
Ovcar-3 long	--	
**Consensus**	--	
ENST00000399410.8 ABCC1-202	CAGCTGGCCCCAGGTGGGCCGAGTGGAATTCCGGAACTACTGCCTGCGCTACCGAGAGGACCTGGACTTC	4060
Caov-3 short	--	
Ovcar-3 short	--	
Caov-3 long	--	
Ovcar-3 long	--	
**Consensus**	--	
ENST00000399410.8 ABCC1-202	GTTCTCAGGCACATCAATGTCACGATCAATGGGGGAGAAAAGGTCGGCATCGTGGGGCGGACGGGAGCTG	4130
Caov-3 short	--	
Ovcar-3 short	--	
Caov-3 long	--	
Ovcar-3 long	--	
**Consensus**	--	
ENST00000399410.8 ABCC1-202	GGAAGTCGTCCCTGACCCTGGGCTTATTTCGGATCAACGAGTCTGCCGAAGGAGAGATCATCATCGATGG	4200
Caov-3 short	--	
Ovcar-3 short	--	
Caov-3 long	--	
Ovcar-3 long	--	

```
Consensus                      ----------------------------------------------------------------------
ENST00000399410.8 ABCC1-202    CATCAACATCGCCAAGATCGGCCTGCACGACCTCCGCTTCAAGATCACCATCATCCCCCAGGACCCTGTT  4270
Caov-3 short                   ----------------------------------------------------------------------
Ovcar-3 short                  ----------------------------------------------------------------------
Caov-3 long                    ----------------------------------------------------------------------
Ovcar-3 long                   ----------------------------------------------------------------------

Consensus                      --------------------------------------------------------NNNAGTCTGGA--G
ENST00000399410.8 ABCC1-202    TTGTTTTCGGGTTCCCTCCGAATGAACCTGGACCCATTCAGCCAGTACTCGGATGAAGAAGTCTGGACGT  4340
Caov-3 short                   ------------------------------------------------------GAGATAGTCTGGA--G  14
Ovcar-3 short                  --------------------------------------------------------AAGAGTCTGGA--G  12
Caov-3 long                    -----------------------------------------------------------AGTCTGGA--G  9
Ovcar-3 long                   -----------------------------------------------------------AGTCTGGG-AG  10

Consensus                      TCCTGGAGCTGGCCCACCTGAAGGACTTCGTGTCAGCCCTTCCTGACAAGCTAGACCATGAATGTGCAGA
ENST00000399410.8 ABCC1-202    CCCTGGAGCTGGCCCACCTGAAGGACTTCGTGTCAGCCCTTCCTGACAAGCTAGACCATGAATGTGCAGA  4410
Caov-3 short                   TCCTGGAGCTGGCCCACCTGAAGGACTTCGTGTCAGCCCTTCCTGACAAGCTAGACCATGAATGTGCAGA  84
Ovcar-3 short                  TCCTGGAGCTGGCCCACCTGAAGGACTTCGTGTCAGCCCTTCCTGACAAGCTAGACCATGAATGTGCAGA  82
Caov-3 long                    TCCTGGAGCTGGCCCACCTGAAGGACTTCGTGTCAGCCCTTCCTGACAAGCTAGACCATGAATGTGCAGA  79
Ovcar-3 long                   TCCTGGAGCTGGCCCACCTGAAGGACTTCGTGTCAGCCCTTCCTGACAAGCTAGACCATGAATGTGCAGA  80

Consensus                      AGGCGGGGAGAACCTCAGTGTCGGGCAGCGCCAGCTTGTGTGCCTAGCCCGGGCCCTGCTGAGGAAGACG
ENST00000399410.8 ABCC1-202    AGGCGGGGAGAACCTCAGTGTCGGGCAGCGCCAGCTTGTGTGCCTAGCCCGGGCCCTGCTGAGGAAGACG  4480
Caov-3 short                   AGGCGGGGAGAACCTCAGTGTCGGGCAGCGCCAGCTTGTGTGCCTAGCCCGGGCCCTGCTGAGGAAGACG  154
Ovcar-3 short                  AGGCGGGGAGAACCTCAGTGTCGGGCAGCGCCAGCTTGTGTGCCTAGCCCGGGCCCTGCTGAGGAAGACG  152
Caov-3 long                    AGGCGGGGAGAACCTCAGTGTCGGGCAGCGCCAGCTTGTGTGCCTAGCCCGGGCCCTGCTGAGGAAGACG  149
Ovcar-3 long                   AGGCGGGGAGAACCTCAGTGTCGGGCAGCGCCAGCTTGTGTGCCTAGCCCGGGCCCTGCTGAGGAAGACG  150

Consensus                      AAGATCCTTGTGTTGGATGAGGCCACGGCAGCCGTGGACCTGGAAACGGACGACCTCATCCAGTCCACCA
ENST00000399410.8 ABCC1-202    AAGATCCTTGTGTTGGATGAGGCCACGGCAGCCGTGGACCTGGAAACGGACGACCTCATCCAGTCCACCA  4550
Caov-3 short                   AAGATCCTTGTGTTGGATGAGGCCACGGCAGCCGTGGACCTGGAAACGGACGACCTCATCCAGTCCACCA  224
Ovcar-3 short                  AAGATCCTTGTGTTGGATGAGGCCACGGCAGCCGTGGACCTGGAAACGGACGACCTCATCCAGTCCACCA  222
Caov-3 long                    AAGATCCTTGTGTTGGATGAGGCCACGGCAGCCGTGGACCTGGAAACGGACGACCTCATCCAGTCCACCA  219
Ovcar-3 long                   AAGATCCTTGTGTTGGATGAGGCCACGGCAGCCGTGGACCTGGAAACGGACGACCTCATCCAGTCCACCA  220

Consensus                      TCCGGACACAGTTCGAGGACTGCACCGTCCTCACCATCGCCCACCGGCTCAACACCATCATGGACTACAC
ENST00000399410.8 ABCC1-202    TCCGGACACAGTTCGAGGACTGCACCGTCCTCACCATCGCCCACCGGCTCAACACCATCATGGACTACAC  4620
Caov-3 short                   TCCGGACACAGTTCGAGGACTGCACCGTCCTCACCATCGCCCACCGGCTCAACACCATCATGGACTACAC  294
Ovcar-3 short                  TCCGGACACAGTTCGAGGACTGCACCGTCCTCACCATCGCCCACCGGCTCAACACCATCATGGACTACAC  292
Caov-3 long                    TCCGGACACAGTTCGAGGACTGCACCGTCCTCACCATCGCCCACCGGCTCAACACCATCATGGACTACAC  289
Ovcar-3 long                   TCCGGACACAGTTCGAGGACTGCACCGTCCTCACCATCGCCCACCGGCTCAACACCATCATGGACTACAC  290
```

**Consensus**	AAGGGTGATCGTCTTGGACAAAGGAGAAATCCAGGAGTACGGCGCCCCATCGGACCTCCTGCAGCAGAGA	
ENST00000399410.8 ABCC1-202	AAGGGTGATCGTCTTGGACAAAGGAGAAATCCAGGAGTACGGCGCCCCATCGGACCTCCTGCAGCAGAGA	4690
Caov-3 short	AAGGGTGATCGTCTTGGACAAAGGAGAAATCCAGGAGTACGGCGCCCCATCGGACCTCCTGCAGCAGAGA	364
Ovcar-3 short	AAGGGTGATCGTCTTGGACAAAGGAGAAATCCAGGAGTACGGCGCCCCATCGGACCTCCTGCAGCAGAGA	362
Caov-3 long	AAGGGTGATCGTCTTGGACAAAGGAGAAATCCAGGAGTACGGCGCCCCATCGGACCTCCTGCAGCAGAGA	359
Ovcar-3 long	AAGGGTGATCGTCTTGGACAAAGGAGAAATCCAGGAGTACGGCGCCCCATCGGACCTCCTGCAGCAGAGA	360

**Consensus**	GGTCTTTTCTACAGCATGGCCAAAGACGCCGGCTTGGTGTGAGCCCCAGAGCTGGCATATCTGGTCAGAA	
ENST00000399410.8 ABCC1-202	GGTCTTTTCTACAGCATGGCCAAAGACGCCGGCTTGGTGTGAGCCCCAGAGCTGGCATATCTGGTCAGAA	4760
Caov-3 short	GGTCTTTTCTACAGCATGGCCAAAGACGCCGGCTTGGTGTGAGCCCCAGAGCTGGCATATCTGGTCAGAA	434
Ovcar-3 short	GGTCTTTTCTACAGCATGGCCAAAGACGCCGGCTTGGTGTGAGCCCCAGAGCTGGCATATCTGGTCAGAA	432
Caov-3 long	GGTCTTTTCTACAGCATGGCCAAAGACGCCGGCTTGGTGTGAGCCCCAGAGCTGGCATATCTGGTCAGAA	429
Ovcar-3 long	GGTCTTTTCTACAGCATGGCCAAAGACGCCGGCTTGGTGTGAGCCCCAGAGCTGGCATATCTGGTCAGAA	430

**Consensus**	CTGCAGGGCCTATATGCCAGCGCCCAGGGAGGAGTCAGTACCCCTGGTAAACCAAGCCTCCCACACTGAA	
ENST00000399410.8 ABCC1-202	CTGCAGGGCCTATATGCCAGCGCCCAGGGAGGAGTCAGTACCCCTGGTAAACCAAGCCTCCCACACTGAA	4830
Caov-3 short	CTGCAGGGCCTATATGCCAGCGCCCAGGGAGGAGTCAGTACCCCTGGTAAACCAAGCCTCCCACACTG--	502
Ovcar-3 short	CTGCAGGGCCTATATGCCAGCGCCCAGGGAGGAGTCAGTACCCCTGGTAAACCAAGCCTCCCACACTGAA	502
Caov-3 long	CTGCAGGGCCTATATGCCAGCGCCCAGGGAGGAGTCAGTACCCCTGGTAAACCAAGCCTCCCACACTGAA	499
Ovcar-3 long	CTGCAGGGCCTATATGCCAGCGCCCAGGGAGGAGTCAGTACCCCTGGTAAACCAAGCCTCCCACACTGAA	500

**Consensus**	ACCAAAACATAAAAACCAAACCCAGACAACCAAAACATATTCAAAGCAGCAGCCACCGCCATCCGGTCCC	
ENST00000399410.8 ABCC1-202	ACCAAAACATAAAAACCAAACCCAGACAACCAAAACATATTCAAAGCAGCAGCCACCGCCATCCGGTCCC	4900
Caov-3 short	--	502
Ovcar-3 short	ACC---	505
Caov-3 long	ACCAAAACATAAAAACCAAACCCAGACAACCAAAACATATTCAAAGCAGCAGCCACCGCCATCCGGTCCC	569
Ovcar-3 long	ACCAAAACATAAAAACCAAACCCAGACAACCAAAACATATTCAAAGCAGCAGCCACCGCCATCCGGTCCC	570

**Consensus**	CTGCCTGGAACTGGCTGTGAAGACCCAGGAGAGACAGAGATGCGAACCACCCAANACACNN-ACACCCTG	
ENST00000399410.8 ABCC1-202	CTGCCTGGAACTGGCTGTGAAGACCCAGGAGAGACAGAGATGCGAACCACCCAAAACACGC-ACACCCTG	4969
Caov-3 short	--	502
Ovcar-3 short	--	505
Caov-3 long	CTGCCTGGAACTGGCTGTGAAGACCCAGGAGAGACAGAGATGCGAACCACCCAAGACACGTGACACCCTG	639
Ovcar-3 long	CTGCCTGGAACTGGCTGTGAAGACCCAGGAGAGACAGAGATGCGAACCACCCAAAACACAC-ACACCCTG	639

**Consensus**	CCCCTGGTGCCCTGAGACAGACACACAGCCTCACG-CCCCCAGGAATGCAAGTGGTTTCCTGGTGCTTCC	
ENST00000399410.8 ABCC1-202	CCCCTGGTGCCCTGAGACAGACACACAGCCTCACG-CCCCCAGGAATGCAAGTGGTTTCCTGGTGCTTCC	5038
Caov-3 short	--	502
Ovcar-3 short	--	505
Caov-3 long	CCCCTGGTGCCCTGAGACAGACACACAGCCTCACGCCCCCCAGGAATGCAAGTGGTTTCCTGGTGCTTCC	709
Ovcar-3 long	CCCCTGGTGCCCTGAGACAGACACACAGCCTCACG-CCCCCAGGAATGCAAGTGGTTTCCTGGTGCTTCC	708

```
CACGGAGGAGTTTTGGCAG CCAGACTTCTGG G T G TTGTATAGA GATCCTAGTGACCAAAT
Consensus                   CACGGAGGAGTTTTGGCAG-CCAGACTTCTGGNNGN--NTNGNTTGTATAGANGATCCTAGTGACCAAAT
ENST00000399410.8 ABCC1-202 CACGGAGGAGTTTTGGCAG-CCAGACTTCTGGAGGA--ATTGGTTGTATAGAAGATCCTAGTGACCAAAT 5105
Caov-3 short                ---------------------------------------------------------------------- 502
Ovcar-3 short               ---------------------------------------------------------------------- 505
Caov-3 long                 CACGGAGGAGTTTTGGCAGCCCAGACTTCTGGAGGACTCTCGTTTGTATAGACGATCCTAGTGACCAAAT 779
Ovcar-3 long                CACGGAGGAGTTTTGGCAG-CCAGACTTCTGGGAGG-AATTGGTTGTATAGAAGATCCTAGTGACCAAAT 776

T CAGCCTACTGCC gGGATCTCTCCAGCCGAAGTCTGT GGACTGCAAGTCTTTGAGATGCTTCTGG
Consensus                   T-CAGCCTACTGCCNN--GGATCTCTCCAGCCGAAGTCTGT-GGACTGCAAGTCTTTGAGATGCTTCTGG
ENST00000399410.8 ABCC1-202 T-CAGCCTACTGCCTC--GGATCTCTCCAGCCGAAGTCTGT-GGACTGCAAGTCTTTGAGATGCTTCTGG 5171
Caov-3 short                ---------------------------------------------------------------------- 502
Ovcar-3 short               ---------------------------------------------------------------------- 505
Caov-3 long                 TCCAGCCTACTGCCCTCGGGATCTCTCCAGCCGAAGTCTGT-GGACTGCAAGTCTTTGAGATGCTTCTGG 848
Ovcar-3 long                T-CAGCCTACTGCCTC-GGGATCTCTCCAGCCGAAGTCTGTGGGACTGCAAGTCTTTGAGATGCTTCTGG 844

CC ATCACCTCTAACATCCTTGTCTGGGTCTACC G A T CATTT C T tGGGG CTG
Consensus                   --NNCCNATCACCTCTAACATCCTTGTCTGGGTCTACCNNGNANNN---TNCATTTNCNT---GGGNCTG
ENST00000399410.8 ABCC1-202 --CTCCCATCACCTCTAACATCCTTGTCTGGGTCTACCAGGAACGC---TTCATTTCCTT---GGGGCTG 5233
Caov-3 short                ---------------------------------------------------------------------- 502
Ovcar-3 short               ---------------------------------------------------------------------- 505
Caov-3 long                 GCTCCCAATCACCTCTAACATCCTTGTCTGGGTCTACCCAGGAACCGCTTCCATTTTCCTTTGGGGCCTG 918
Ovcar-3 long                --CTCCCATCACCTCTAACATCCTTGTCTGGGTCTACCAGGGAACG--CTTCATTTCCCT-TGGGGGCTG 909

CAGTTTT gG G GGG C G AGAAAATCATTTT CC T GGCAGTGTCCCAGGGCCC
Consensus                   CAGTTTT-----GNNNNNGNGGGNCNNGNAGAAAATCATTTTN---------------------------
ENST00000399410.8 ABCC1-202 CAGTTTT-----GTGGTTGAGGGGCCTGGAGAAAATCATTTTCTCCCCTT--GGCAGTGTCCCAGGGCCC 5296
Caov-3 short                ---------------------------------------------------------------------- 502
Ovcar-3 short               ---------------------------------------------------------------------- 505
Caov-3 long                 CAGTTTTTGTGGGTTGGAGGGGGCCTGGGAGAAAATCATTTTC--------------------------- 961
Ovcar-3 long                CAGTTTT---GTGGGTTTGAGGGCCCTGTAGAAAATCATTTTTCTCCTCTTGGGCAGTGTCCCAGGGCCC 976

T GATGGT CTCTTACCAACATCTGGTCTTC G CTCA AAGCTGGGA C AGCATCTCAGCGCCAG
Consensus                   ----------------------------------------------------------------------
ENST00000399410.8 ABCC1-202 TGGATGGTCCTCTTACCAACATCTGGTCTTCCAGGCACTCAAAAGCTGGGAACCAGCATCTCAGCGCCAG 5366
Caov-3 short                ---------------------------------------------------------------------- 502
Ovcar-3 short               ---------------------------------------------------------------------- 505
Caov-3 long                 ---------------------------------------------------------------------- 961
Ovcar-3 long                T-GATGGT-CTCTTACCAACATCTGGTCTTCAGGCACCTCAGAAGCTGGGATCAAGCATCTCAGCGCCAG 1044

CTCTA CAGTTCTCGTT
Consensus                   ----------------------------------------------------------------------
ENST00000399410.8 ABCC1-202 CTCTACCAGTTCTCGTTTTGGGCCAGAGGCAGCCTCTGCACTCCCACGCCTGTCCTCCTGGAAGGGACCT 5436
Caov-3 short                ---------------------------------------------------------------------- 502
Ovcar-3 short               ---------------------------------------------------------------------- 505
Caov-3 long                 ---------------------------------------------------------------------- 961
Ovcar-3 long                CTCTA-CAGTTCTCGTT----------------------------------------------------- 1060
```

```
Consensus                    ----------------------------------------------------------------------
ENST00000399410.8 ABCC1-202  GGTTGGACTAACGGCTAACCTGGACCTGGAACTGTAGGGCCAGGGGATTGTCTCAGGGCCGACGTTCCAC 5506
Caov-3 short                 ---------------------------------------------------------------------- 502
Ovcar-3 short                ---------------------------------------------------------------------- 505
Caov-3 long                  ---------------------------------------------------------------------- 961
Ovcar-3 long                 ---------------------------------------------------------------------- 1060
```

```
Consensus                    ----------------------------------------------------------------------
ENST00000399410.8 ABCC1-202  CTGGGGCTTCCCTCCCCACCCACCCCGACTCCAGGCTTTCCCTTTTTTCTTTTGTTCAACATTGTAAGAA 5576
Caov-3 short                 ---------------------------------------------------------------------- 502
Ovcar-3 short                ---------------------------------------------------------------------- 505
Caov-3 long                  ---------------------------------------------------------------------- 961
Ovcar-3 long                 ---------------------------------------------------------------------- 1060
```

```
Consensus                    ----------------------------------------------------------------------
ENST00000399410.8 ABCC1-202  CAATCAATGCTGTTATTACTGTTCCCACCATGATTGATGTGGGGTAAATATTAAGGAGATGGCCTCATGG 5646
Caov-3 short                 ---------------------------------------------------------------------- 502
Ovcar-3 short                ---------------------------------------------------------------------- 505
Caov-3 long                  ---------------------------------------------------------------------- 961
Ovcar-3 long                 ---------------------------------------------------------------------- 1060
```

```
Consensus                    ----------------------------------------------------------------------
ENST00000399410.8 ABCC1-202  GAATTTGACCTTGACTAGAAATAGAGACTGAGAGTGAGCAACCAGCTGGAAGGTACTATGCCAGTCCTAG 5716
Caov-3 short                 ---------------------------------------------------------------------- 502
Ovcar-3 short                ---------------------------------------------------------------------- 505
Caov-3 long                  ---------------------------------------------------------------------- 961
Ovcar-3 long                 ---------------------------------------------------------------------- 1060
```

```
Consensus                    ----------------------------------------------------------------------
ENST00000399410.8 ABCC1-202  CAGAAAAATGTGTTAGGGGCCTGGCCCAAAGCAGTGTTGGTTGCTTACAGTGTTGATTGATTTTGTTCTT 5786
Caov-3 short                 ---------------------------------------------------------------------- 502
Ovcar-3 short                ---------------------------------------------------------------------- 505
Caov-3 long                  ---------------------------------------------------------------------- 961
Ovcar-3 long                 ---------------------------------------------------------------------- 1060
```

```
Consensus                    ----------------------------------------------------------------------
ENST00000399410.8 ABCC1-202  TTTTCTTACCACCTCTTTTCTTTCCCTCTCATGGTACCTGCTCATGGTTATGAAGCTTTCAAAGTAAAGA 5856
Caov-3 short                 ---------------------------------------------------------------------- 502
Ovcar-3 short                ---------------------------------------------------------------------- 505
Caov-3 long                  ---------------------------------------------------------------------- 961
Ovcar-3 long                 ---------------------------------------------------------------------- 1060
```

```
Consensus                      ----------------------------------------------------------------------
ENST00000399410.8 ABCC1-202    ACACGAAATACCTCCCAAGTATTACCAGTGGGTACCAAAAAAATGTCCCCTTGAGTCTTTTCCTTGTTTT  5926
Caov-3 short                   ----------------------------------------------------------------------  502
Ovcar-3 short                  ----------------------------------------------------------------------  505
Caov-3 long                    ----------------------------------------------------------------------  961
Ovcar-3 long                   ----------------------------------------------------------------------  1060
```

```
Consensus                      ----------------------------------------------------------------------
ENST00000399410.8 ABCC1-202    TAGATGTTAATTCTCTCCCTTGGCATCCGGTTAGCCCCCCAGGGGGGGCAGCATTGTGGAGAACTTGATA  5996
Caov-3 short                   ----------------------------------------------------------------------  502
Ovcar-3 short                  ----------------------------------------------------------------------  505
Caov-3 long                    ----------------------------------------------------------------------  961
Ovcar-3 long                   ----------------------------------------------------------------------  1060
```

```
Consensus                      ----------------------------------------------------------------------
ENST00000399410.8 ABCC1-202    TTTAGTTACTGATGCTCTTCCAGGACACGAAAAGAACCCATCTTTGAATATCAATGATTTTTTTTTTTTA  6066
Caov-3 short                   ----------------------------------------------------------------------  502
Ovcar-3 short                  ----------------------------------------------------------------------  505
Caov-3 long                    ----------------------------------------------------------------------  961
Ovcar-3 long                   ----------------------------------------------------------------------  1060
```

```
Consensus                      ----------------------------------------------------------------------
ENST00000399410.8 ABCC1-202    AGTACTGTTCCGGGGAGAAAAACAGTCTCAAAACTTGAACTTCTTGGGAATAGAAGTGTTGGGCTGAGAA  6136
Caov-3 short                   ----------------------------------------------------------------------  502
Ovcar-3 short                  ----------------------------------------------------------------------  505
Caov-3 long                    ----------------------------------------------------------------------  961
Ovcar-3 long                   ----------------------------------------------------------------------  1060
```

```
Consensus                      ----------------------------------------------------------------------
ENST00000399410.8 ABCC1-202    GTAACATTCCCAGGAAATAGTGAGAAGCTCGCCCTGTGTTTGAAACCGTGTTGGTCTCTGTGTTCCTGGA  6206
Caov-3 short                   ----------------------------------------------------------------------  502
Ovcar-3 short                  ----------------------------------------------------------------------  505
Caov-3 long                    ----------------------------------------------------------------------  961
Ovcar-3 long                   ----------------------------------------------------------------------  1060
```

```
Consensus                      ----------------------------------------------------------------------
ENST00000399410.8 ABCC1-202    AGAAAACAGGGAAGCAGCATCTTTTAAAGCCTGTTCTTTAAGGTGTCTCGTTAGAGCCCAAAGTGGAATC  6276
Caov-3 short                   ----------------------------------------------------------------------  502
Ovcar-3 short                  ----------------------------------------------------------------------  505
Caov-3 long                    ----------------------------------------------------------------------  961
Ovcar-3 long                   ----------------------------------------------------------------------  1060
```

```
Consensus                     ----------------------------------------------------------------------
ENST00000399410.8 ABCC1-202   CGGAAGGCAGCCAGAGCTGAGGCTGCCCCAAGACTCAGACTTGCTAAGAATTACGCCGCCGACTTCAAAC  6346
Caov-3 short                  ----------------------------------------------------------------------  502
Ovcar-3 short                 ----------------------------------------------------------------------  505
Caov-3 long                   ----------------------------------------------------------------------  961
Ovcar-3 long                  ----------------------------------------------------------------------  1060
```

```
Consensus                     ----------------------------------------------------------------------
ENST00000399410.8 ABCC1-202   CCAGAGAGCATCTTTCTTTTAGGCGAAAACGCATATATTTATTTTTTGTAAGTTATACCATTCTTTCACA  6416
Caov-3 short                  ----------------------------------------------------------------------  502
Ovcar-3 short                 ----------------------------------------------------------------------  505
Caov-3 long                   ----------------------------------------------------------------------  961
Ovcar-3 long                  ----------------------------------------------------------------------  1060
```

```
Consensus                     ----------------------------------------------------------------------
ENST00000399410.8 ABCC1-202   TTAGATAAACTAAGTTTTGGGGGATCCTTTTGTAATGACTTACACTGGAAATGCGAACATTTGCAGTAAA  6486
Caov-3 short                  ----------------------------------------------------------------------  502
Ovcar-3 short                 ----------------------------------------------------------------------  505
Caov-3 long                   ----------------------------------------------------------------------  961
Ovcar-3 long                  ----------------------------------------------------------------------  1060
```

```
Consensus                     ------------------
ENST00000399410.8 ABCC1-202   AAAATATATATATATCTA  6504
Caov-3 short                  ------------------  502
Ovcar-3 short                 ------------------  505
Caov-3 long                   ------------------  961
Ovcar-3 long                  ------------------  1060
```

S**upplementary Figure 3.** Complete sequence alignment of our sequences with ENST00000399410.8. ABCC1-202. We used MAFFT SnapGene software to align our sequences to the ABCC1 transcript ENST000000399410.8. Shown are the short and long sequences derived from 3'RACE and Sanger Sequencing for the Caov-3 and Ovcar3- cells.

Sequence Logo: 50% GC base composition

Consensus Threshold: >50%

Compare to: the consensus

Bases that match the reference are marked with yellow highlighting.

Description:

Ensembl genome ENST00000399410.8 ABCC1-202
NCBI NM_004996.4

File 1. CA2.4B short form 100 (600 with ORF)
File 2. OV2.4D short form 100 (600 with ORF)
File 3. Ca2.1D long form 1600
File 4. Ova2.1B long form 1600

Created: Aug 17, 2022

Last Modified: Aug 17, 2022
